# Effect of differences in residual feed intake on gastrointestinal microbiota of Dexin fine-wool meat sheep

**DOI:** 10.3389/fmicb.2024.1482017

**Published:** 2024-12-20

**Authors:** Ziting Wang, Weiwei Wu, Xuefeng Lv, Weiting Xing, Xu Wang, Yong Tuo, Yan Ma, Linjiao He, Zhijun Zhang, Wenxin Zheng

**Affiliations:** ^1^Feed Research Institute of Xinjiang Academy of Animal Husbandry Sciences, Urumqi, China; ^2^College of Animal Science, Xinjiang Agricultural University, Urumqi, China; ^3^Xinjiang Feed Biotechnology Key Laboratory, Urumqi, China; ^4^Xinjiang Academy of Animal Husbandry Sciences, Urumqi, China

**Keywords:** fine-wool sheep, bacteria, fungi, gastrointestinal tract, residual intake feed

## Abstract

In this study, we examined the effects of different residual feed intakes (RFIs) on nutrient digestibility and the microbiota of the digestive tract of Dexin fine-wool sheep. Fifty 70-day-old Dexin fine-wool meat lambs were selected as the experimental group and fed in a single pen for 100 days. Based on their mid-term metabolic weight, 100-day average daily weight gain and daily feed intake, the male Dexin lambs were divided into a low-RFI group (13), a mid-RFI group (18), and a high-RFI group (11). Six male Dexin lambs were selected from each group to collect feces, rumen digesta and solid digesta. Rectal feces were collected from three lambs in each group. The results showed that the digestibility of dry matter and crude protein by sheep in the L-RFI group was than that in the H-RFI group (*P* < 0.05). Within the microbial population, f_Anaerovoracaceae, g_Christensenellaceae_R_7_group, p_Proteobacteria, and g_Roseburia were significantly correlated with RFI. Energy metabolism, metabolism of amino acids and carbohydrates, transport and catabolism, and cell migration pathways were upregulated in the L-RFI group. The differences in the microbiota of the digestive tract of sheep with different RFIs were reflected in the presence of some key bacterial genera rather than changes in the overall microbial diversity.

## 1 Introduction

The rumen is a digestive organ unique to ruminants. It has a distinctive microbial fermentation system that can efficiently convert macromolecular substances from the diet, such as lignin, cellulose, and non-protein nitrogen, into nutrients that are easier for the host to utilize (Krautkramer et al., 2021). Early research focused on the structure of the complex microbial community in the rumen and its impact on nutrient usage from feeds of different compositions. These microorganisms metabolize dietary compounds and produce volatile fatty acids (VFAs), amino acids and other essential nutrients, thereby providing energy for the host body and maintaining the optimal functioning of the rumen (Tellez et al., 2006).

The hindgut of ruminants was previously considered the endpoint of digestion; however, in-depth research on the fungal microbiota of ruminants has shown that the fungi in the rectum produce a large number of lignin-degrading enzymes that can ferment unused lignin. These enzymes effectively decomposed the structural polymers of plant cell walls, thereby improving the absorption and utilization rate of their nutrients (Du et al., 2023).

Compared with the traditional measurement of feed conversion ratio (FCR), residual feed intake (RFI) is considered to be a more accurate and flexible assessment method. RFI refers to the difference between the actual feed intake of an individual animal and the expected feed intake based on its body size and production performance. Low RFI indicates that individual animals consume less feed than predicted and therefore have a lower environmental pollution capacity, without affecting individual body weight, daily weight gain, or body shape.

In studies on the microbiota of ruminants with different RFIs, Herd and Arthur (2009) and Paz et al. (2018) reported that rumen fermentation patterns and the microbial composition of the ruminant gut accounted for 19%-20% of the variation in RFI. Ellison et al. (2019) found that six types of rumen microorganisms were highly correlated with actual feed efficiency in ewes. Liu et al. (2022) showed that the abundance of *Firmicutes* and *Bacteroidetes* in the intestines of Angus cattle with low RFI was significantly higher than that of cattle with high RFI. Elolimy et al. (2020) found that the abundance of *Bacteroidota* in rectal feces of Holstein cattle was higher in low-RFI cattle than in high-RFI cattle. Both *Firmicutes* and *Bacteroidetes* are the most abundant bacterial groups in the digestive tract of ruminants and play a major role in fiber fermentation in the diet.

Previous studies have shown that the gastrointestinal microbiota of ruminants affects RFI expression, which is one of the important indicators of feed efficiency type. Digestion and absorption in ruminants are processes that involve a number of interconnected steps in the gastrointestinal tract (GIT). For our experiments, we chose Dexin fine-wool meat sheep of the same age and under the same feeding conditions. To systematically examine the influence of RFI on the digestive tract as a whole, the type and abundance of microorganisms in the rumen, ileum and rectum were determined to represent the overall GIT of the sheep under different feed efficiencies.

## 2 Materials and methods

All animal procedures were approved by the Agreement Management and Review Committee of the Feed Research Institute of the Xinjiang Academy of Animal Sciences (Approval No. 2 20230620).

### 2.1 Animals and experimental period

**Figure 1:**
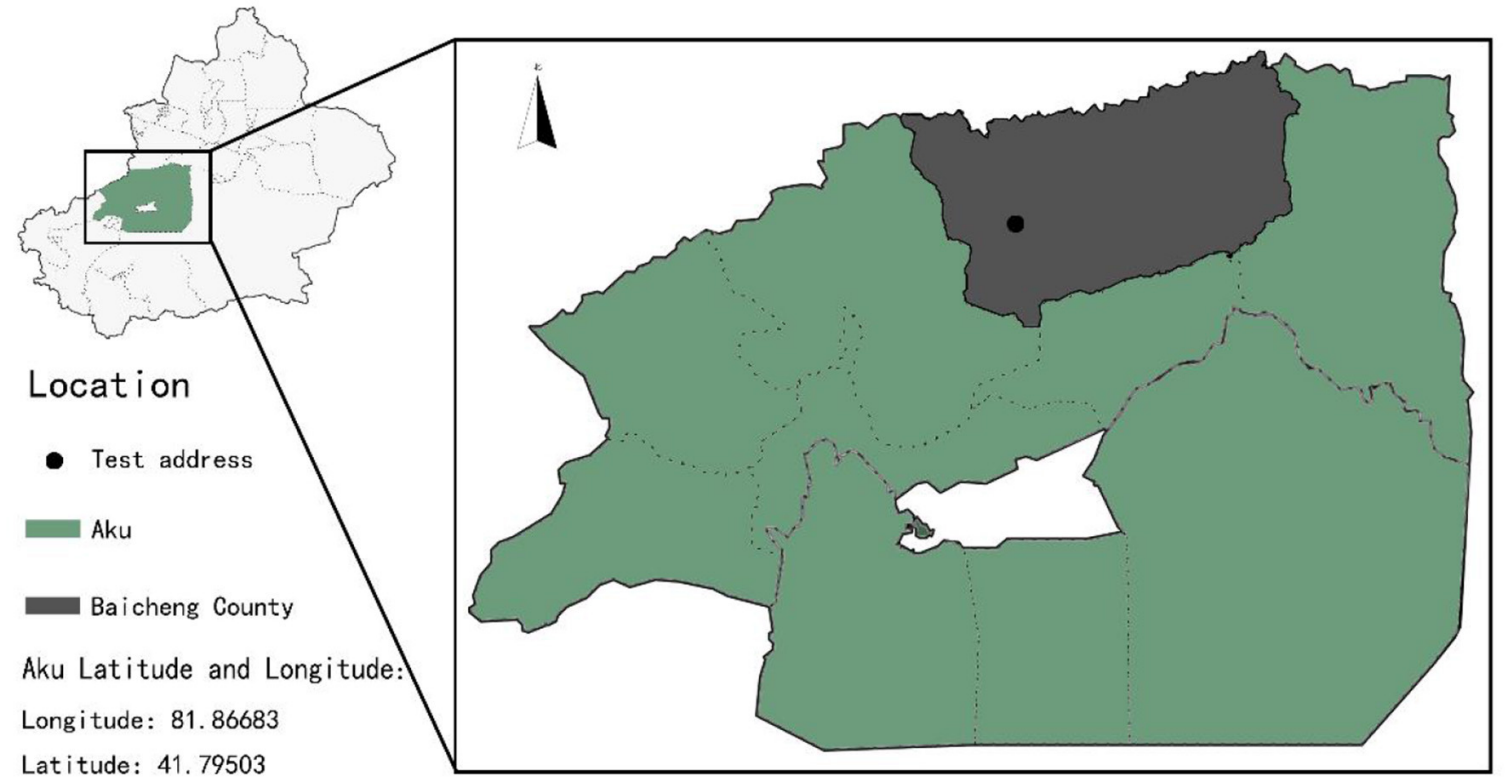
Test location at the Baicheng County Breeding Sheep Farm in Xinjiang, China.

The test was conducted at the Baicheng County Breeding Sheep Farm in Xinjiang, China (Figure 1). Fifty 70-day-old Dexin fine-wool meat sheep were purchased from the Baicheng County Breeding Sheep Farm. Deworming was performed twice, and the sheep were weighed before the experiment started (30.77 ± 3.17 kg). The trial period lasted 100 days, including a 14-day pre-feeding period. The animals were weighed every 20 days during the trial period and slaughtered on the 100^th^ day of the trial period.

### 2.2 Feed formulation and processing

The feed formula selected was the 678S-2 experimental pelleted feed produced by Tiankang Feed Technology Co., Ltd. The nutrient levels in the feed are shown in Table 1.

**Table 1:** Percent composition and nutrient levels of basal diets (air-dried basis).

**Items**	**Content**
**Ingredients**	
Corn stover	13.40
Alfalfa powder	14.35
Soybean meal	11.48
Cottonseed meal	12.44
Corn	38.00
Flour	6.70
Rock flour	1.0
Calcium bicarbonate	0.19
Sodium bicarbonate	0.77
Sodium chloride	1.0
Premix^a^	0.67
**Nutrient levels** ^b^	
DM	87.10
CP	16.17
EE	1.83
Ash	11.65
NDF	36.89
ADF	29.85
Ca	0.69
P	0.39
ME MJ/kg	15.71

^a^One kilogram of premix contained the following: VA 150,000 IU, VD_3_ 56,500 IU, VE 8,000 IU, Se (as sodium selenite) 14 mg, I (as Potassium iodide) 80 mg, Cu (as coPPer sulfate) 290 mg, Mn (as manganese sulfate) 1,925 mg, Zn (as zinc oxide) 2,050 mg, Co (as cobalt sulfate) 24 mg.

^b^ME was a calculated value, while the others were measured values.

### 2.3 Experiment management

Before the test, the pen was thoroughly disinfected and the sheep entering the pen were marked with numbered ear tags. Before the start of the pilot test period, the experimental animals were dewormed using a combination of intramuscular injection and feeding of anthelminthics. Sheep in individual cages were fed twice a day at 10:00 and 18:00, with free access to food and water, ensuring that the amount of leftover feed for each sheep was >15% per day. During the experiment, three test sheep were eliminated due to disease, and five test rams were selected for breeding.

### 2.4 Measurement indices and methods

#### 2.4.1 Calculation and grouping of residual feed intake

Using SPSS26.0 for analysis, the R^2^ values of dry matter feed intake, daily weight gain and average mid-term metabolic weight of the test sheep met the conditions of the regression equation. A linear model was constructed to calculate the RFI of the test sheep as follows:


$$\begin{array}{l} \text{Yi}=\text{β}0+\text{β}1\left(\text{ADGi}\right)+\text{β}2\text{MBWi}+\text{ei} \end{array}$$

The average daily weight gain, ADGi = (FBWi – IBWi)/N was also calculated. FBWi (final body weight) is the weight of individual *i* at the end of the trial, IBWi (initial body weight) is the initial weight of individual *i*, and N is the number of trial days. The formula for average metabolic body weight is MBWi = [1/2 × (FBWi + IBWi)] 0.75, where β0 is the regression intercept, β1 and β2 are fixed values, and *ei* is the RFI of sheep *i* (Koch et al., 1963).

Based on the mean and standard deviation of the RFI, the test sheep were divided into a high RFI (H-RFI) group (RFI > mean + 0.5SD) with 11 sheep, a medium RFI (M-RFI) group (mean + 0.5SD < RFI < mean – 0.5SD) with 18 sheep, and a low RFI (L-RFI) group (RFI < mean – 0.5SD) with 13 sheep (Zhang, 2019).

#### 2.4.2 Sample collection

On the 90^th^ day of the experimental period, six lambs from each group were selected to collect feces, twice a day for 3 days. The samples were placed in plastic bags and stored at −20°C until used. On the 100^th^ day of the experimental period, the animals were humanely slaughtered before feeding. Six Dexin male lambs were randomly selected from each group and 10 ml of solid digesta from the rumen and the ileum solid digesta were taken, transferred to cryopreservation tubes, and stored in liquid nitrogen. During the collection process, there was no chyme or feces in the rectum of some slaughtered sheep, so only the rectal feces of three lambs from each group were collected and stored in liquid nitrogen. The rumen chyme, ileum chyme and rectal feces were sent to the Xinjiang Morgan Biotechnology Co., Ltd. for sequencing analysis of the bacterial and fungal microbiota.

#### 2.4.3 Measurement of GIT indices and data analysis

Apparent digestibility was determined according to a published method using hydrochloric acid-insoluble ash content of feces and feeds as a readout (Van Keulen, 1977):


$$\begin{array}{l} \text{Nutrient apparent digestibility}=\text{100}\\ \times [\text{1}-(\text{HCl}-\text{insoluble ash in feed/HCl}-\text{insoluble ash in feces})\\ \times (\text{nutrient content in feces/nutrient content in feed})] \end{array}$$

The nutrient digestibility of dry matter, crude protein and neutral detergent fiber was also calculated.

Ammoniacal nitrogen was determined using indophenol blue colorimetry according to a published method (Li, 2015). Concentrations of volatile fatty acids were determined by gas chromatography. Briefly, VFAs were separated on a 2 m glass column (3 mm i.d.) using a Fisons HRGC MEGA 2 Series model 8560 chromatograph (Fison Instruments, Glasgow, UK) equipped with a flame ionization detector (Fison Instruments, Glasgow, UK). The chromatographic column was 10% SP-1000 + 1% H3PO4, the chromatographic column was 100/120 ChromosorbWAW (Tehnokroma Analitica SA, Sant Cugat del Valles, Spain), and the carrier gas was nitrogen. The injector and detector temperatures were 200°C, and the column temperature was 155°C. The internal standard used was 2-ethylbutyric acid (Sigma Aldrich, Taufkirchen, Germany). GC was used to determine the concentration of acetic acid, propionic acid, butyric acid, and valeric acid in the rumen.

For determining the genus and species of microbiota in the rumen, genomic DNA rumen fluid sample was extracted by the cetyltrimethylammonium bromide (CTAB) method. DNA concentration and purity was determined by 0.8% agarose gel electrophoresis, and the DNA was diluted to 1 ng/μL for use. PCR amplification was performed using the V3 region-specific primers of 16S rDNA, the extracted DNA, a fungal ITS1 primer pair (F - 5′CTTGGTCATTTAGAGGAGTAA3 and R - 5′GCTGGTTTCTTTCATATCGATTGCB), and the appropriate combination of PCR reagents and polymerase for amplification.

The PCR products were run on an agarose gel and isolated with an OmegaDNA purification kit (Omega Corporation, USA). The purified PCR products were collected and sequenced on an IlluminaNovaSeq. PCR amplification, paired-end sequencing on the 6,000 platform, Illumina HiSeq sequencing, and analysis of the results were performed by the Beijing NuoHe Bioinformation Technology Co., Ltd. Amplicon qiime2 cloud platform (https://magic-plus.novogene.com/ assessed on 16 September 2023).

## 3 Results

### 3.1 Differences in growth performance of male Dexin lambs with different RFI

As shown in Table 2, the daily feed intake and RFI in the H-RFI group were significantly higher than those in the L-RFI and the M-RFI groups (*P* < 0.01). The F/G was significantly higher than that of the L-RFI and M-RFI groups (*P* < 0.05), and there was no significant difference in the daily weight gain and mid-term metabolic weight of the sheep (*P* < 0.05).

**Table 2:** Differences in growth performance of male Dexin lambs with different RFI.

**Items**	**Group**			**SEM**	***P*-value**
	**L-RFI**	**M-RFI**	**H-RFI**		
The number of animals	13	18	11		
DMI (kg/d)	1.61^B^	1.68^B^	1.83^A^	0.02	0.001
ADG (kg/d)	0.314	0.316	0.331	0.01	0.73
Mid-term metabolic weight (kg)	15.13	14.77	15.24	0.16	0.47
Initial weight (kg)	31.16	29.96	31.18	0.50	0.52
Final weight (kg)	43.73	42.62	44.41	0.62	0.51
F/G	5.03^b^	5.16^b^	5.54^a^	0.07	0.01
RFI	−0.13^Cc^	0.00^Bb^	0.11^Aa^	0.01	0.001

In the same row, values with no letter or the same letter superscripts mean no significant difference (*P* > 0.05), while with different small letter superscripts mean significant difference (*P* < 0.05), and with different capital letter superscripts mean extremely significant difference (*P* < 0.01). ADG, Average daily weight gain; DMI, Dry matter intake; F/G, Feed conversion rate; RFI, Residual feed intake.

### 3.2 Differences in apparent digestibility and rumen fermentation parameters of male Dexin lambs with different RFIs

**Figure 2:**
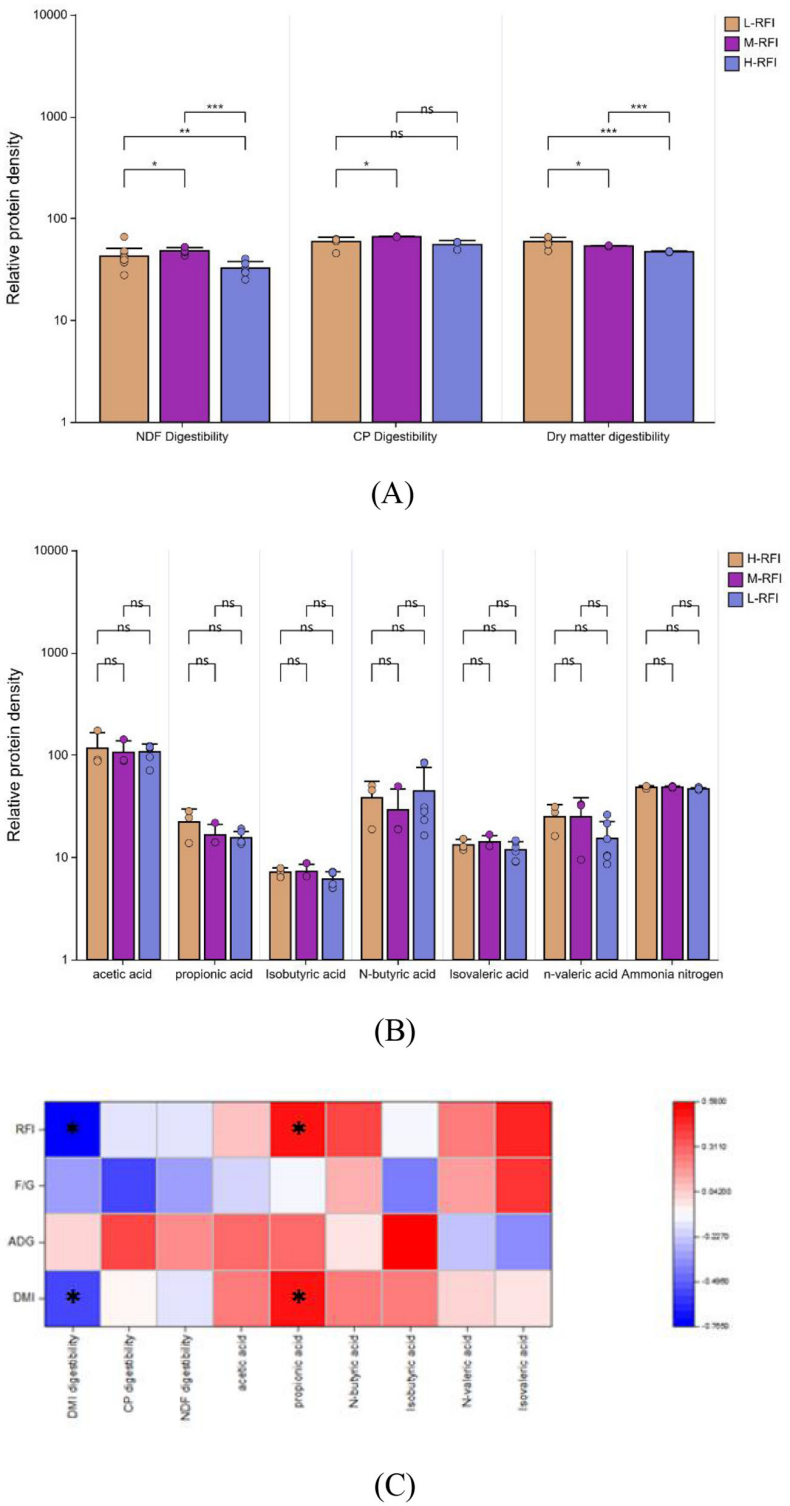
Differences in apparent digestibility **(A)** and ruminal fermentation parameters **(B)** of male Dexin lambs with different RFIs **(C)**. *, ** represents significant, and *** represents extremely significant.

As shown in Figure 2A, the apparent digestibility of dry matter in the L-RFI group was extremely significantly higher than the H-RFI group (*P* < 0.01) and significantly higher than the M-RFI group (*P* < 0.05), the M-RFI group was extremely significantly higher than the H-RFI group(*P* < 0.01), and the apparent digestibility of crude protein in the L-RFI group was significantly higher than M-RFI group (*P* < 0.05), and the apparent digestibility of neutral detergent fiber the L-RFI group was significantly higher than the M-RFI group and the H-RFI group (*P* < 0.05), the M-RFI group was extremely significantly higher than the H-RFI group(*P* < 0.01). There was no significant difference in ammonia nitrogen and volatile fatty acids between lambs with different RFI (*P* > 0.05) (Figure 2B). RFI and DMI were significantly negatively correlated with dry matter digestibility (r = −0.765, −0.546) and significantly positively correlated with propionic acid (r = 0.518, 0.500). ADG was significantly positively correlated with isobutyric acid (r = 0.578) (Figure 2C).

### 3.3 Differences in rumen microorganisms of Dexin male lambs with different RFIs

As shown in Supplementary Figure S1A, in the analysis of rumen chyme samples of Dexin lambs with different RFIs, 4,160 bacterial OTUs were found in the L-RFI group, 1,359 OTUs in the M-RFI, and 3,872 OTUs in the H-RFI. Among them, there were 845 OTUs in common in the three groups, 1,078 OTUs in common in L-RFI and M-RFI, 1,698 OTUs in common in L-RFI and H-RFI, and 1,081 OTUs in common in H-RFI and M-RFI. With respect to the fungi, 775 OTUs were found in L-RFI, 934 OTUs were found in M-RFI, and 747 OTUs were found in H-RFI (Supplementary Figure S1B). Among them, L-RFI and H-RFI had 97 OTUs in common, L-RFI and M-RFI had 115, M-RFI and H-RFI had 102, and among all three groups there were 63 OTUs in common.

As shown in Supplementary Tables S1, S2, there were no significant differences in Chao1, Shannon, and Simpson indices of bacteria and fungi in rumen digesta among male Dexin lambs with different RFIs (*P* > 0.05).

As shown in Supplementary Figures S2A, B, the PCoA plots of rumen digesta of male Dexin lambs with different RFIs partially overlapped without obvious separation, indicating that the differences in microbial communities between and within rumen fluid sample groups were small.

**Figure 3:**
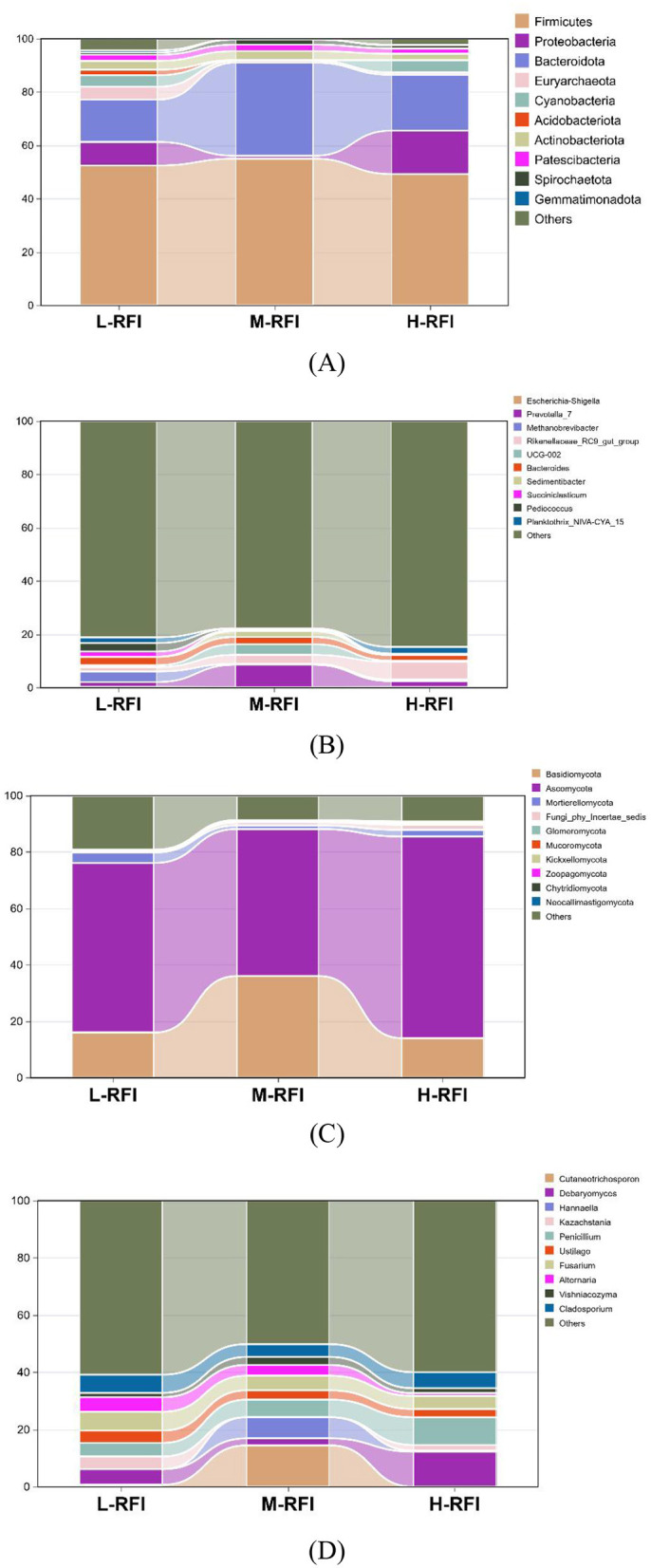
Histogram of bacterial microbiota in the rumen fluid of male Dexin lambs with different RFIs. Bacterial phylum level **(A)**, Bacterial genus level **(B)**, Fungal phylum level **(C)**, and Fungal genus level **(D)**.

As shown in Figure 3A and Table 3, at the phylum level, *Firmicutes, Bacteroidia*, and *Proteobacteria* were the dominant bacterial groups in rumen fluid, The *Bacteroidota* bacteria in the L-RFI group were significantly lower than those in the M-RFI group(*P* < 0.05), while there were no significant differences in the other bacteria (*P* > 0.05). At the genus level, *Escherichia-Shigella, Prevotella*_7 and *Methanobrevibacter* were the dominant bacterial genera in rumen fluid, and there was no significant difference among the top ten bacterial genera (*P* > 0.05) (Figure 3B, Table 4). At the fungal phylum level, *Ascomycota, Basidiomycota*, and *Mortierellomycota* were the dominant phyla, and there was no significant difference among the top ten fungal phyla groups (*P* > 0.05) (Figure 3C, Table 5). At the fungal genus level, *Cladosporium, Fusarium*, and *Debaryomyces* were the dominant genera, and there was no significant difference among the top ten fungal genera (*P* > 0.05) (Figure 3D, Table 6).

**Table 3:** Relative abundance of bacterial microbiota at the phylum level in ruminal fluid from different RFI groups.

**Item**	**Group**			**SEM**	***P*-value**
	**L-RFI**	**M-RFI**	**H-RFI**		
*Firmicutes*	52.47 ± 17.05	55.17 ± 7.88	49.01 ± 11.34	2.87	0.71
*Proteobacteria*	8.81 ± 1.29	1.07 ± 1.09	16.19 ± 2.15	3.40	0.17
*Bacteroidota*	15.90 ± 11.82^b^	35.02 ± 9.20^a^	20.81 ± 16.28^ab^	3.40	0.05
*Euryarchaeota*	4.81 ± 1.05	0.73 ± 0.08	0.87 ± 0.7	1.42	0.44
*Cyanobacteria*	4.32 ± 0.66	0.23 ± 0.03	4.52 ± 0.64	1.27	0.32
*Acidobacteriota*	2.05 ± 0.48	0.003 ± 0.001	0.05 ± 0.009	0.07	0.45
*Actinobacteriota*	3.33 ± 0.32	3.35 ± 0.28	2.43 ± 0.17	0.06	0.79
*Patescibacteria*	2.28 ± 0.33	2.41 ± 0.29	1.96 ± 0.11	0.10	0.95
*Spirochaetota*	0.83 ± 0.06	1.94 ± 0.19	1.22 ± 0.13	0.06	0.39
*Gemmatimonadota*	0.85 ± 0.20	0.008 ± 0.001	0.19 ± 0.03	0.03	0.46
Others	4.30 ± 0.62	0.27 ± 0.03	2.20 ± 0.20	0.09	0.21

In the same row, values with no letter or the same letter superscripts mean no significant difference (*P* > 0.05), while with different small letter superscripts mean significant difference (*P* < 0.05).

**Table 4:** Relative abundance of bacteria and microorganisms at the genus level in rumen digesta of male Dexin lambs with different RFIs.

**Item**	**Group**			**SEM**	***P*-value**
	**L-RFI**	**M-RFI**	**H-RFI**		
*Escherichia-Shigella*	0.32 ± 0.31	0.19 ± 0.30	0.27 ± 0.25	0.08	0.83
*Prevotella_7*	1.84 ± 2.53	8.45 ± 10.05	2.00 ± 2.38	1.55	0.14
*Methanobrevibacter*	3.96 ± 9.01	0.36 ± 0.52	0.58 ± 0.67	1.22	0.42
*Rikenellaceae_RC9_gut_group*	1.64 ± 2.35	3.33 ± 4.17	5.99 ± 7.28	1.19	0.34
*UCG-002*	0.71 ± 0.74	4.02 ± 6.76	0.38 ± 0.56	0.95	0.24
*Bacteroides*	3.12 ± 4.88	2.69 ± 5.41	1.92 ± 0.90	0.95	0.88
*Sedimentibacter*	0.11 ± 0.24	2.20 ± 5.32	0.05 ± 0.06	0.72	0.40
*Succiniclasticum*	1.93 ± 3.87	0.41 ± 0.47	0.09 ± 0.07	0.53	0.34
*Pediococcus*	3.16 ± 4.09	0.61 ± 0.67	0.37 ± 0.86	0.62	0.12
*Planktothrix_NIVA-CYA_15*	2.14 ± 3.51	0.07 ± 0.12	2.28 ± 3.72	0.03	0.32
Others	81.06 ± 15.38	77.68 ± 8.12	76.96 ± 18.41	3.26	0.87

**Table 5:** Relative abundance of fungi at the phylum level in the rumen of male Dexin lambs with different RFIs.

**Item**	**Group**			**SEM**	***P*-value**
	**L-RFI**	**M-RFI**	**H-RFI**		
*Basidiomycota*	16.03 ± 1.41	36.05 ± 3.47	14.04 ± 7.79	5.46	0.19
*Ascomycota*	60.08 ± 22.24	52.12 ± 20.16	71.45 ± 8.15	5.26	0.34
*Mortierellomycota*	3.72 ± 1.44	1.24 ± 0.85	2.36 ± 2.12	3.40	0.35
*Fungi_phy_Incertae_sedis*	0.18 ± 0.03	1.13 ± 0.13	1.87 ± 0.22	0.37	0.18
*Glomeromycota*	0.51 ± 0.09	0.27 ± 0.05	0.29 ± 0.07	1.27	0.82
*Mucoromycota*	0.00	0.03 ± 0.003	0.43 ± 0.07	0.11	0.20
*Kickxellomycota*	0.24 ± 0.005	0.00	0.00	0.79	0.39
*Zoopagomycota*	0.00	0.23 ± 0.05	0.00	0.77	0.39
*Chytridiomycota*	0.02 ± 0.003	0.27 ± 0.05	0.27 ± 0.05	0.09	0.46
*Neocallimastigomycota*	0.05 ± 0.01	0.00	0.16 ± 0.04	0.05	0.49
Others	19.17 ± 2.51	8.68 ± 5.85	9.14 ± 6.64	3.60	0.43

**Table 6:** Relative abundance of fungi at the genus level in rumen digesta of male Dexin lambs with different RFIs.

**Item**	**Group**			**SEM**	***P*-value**
	**L-RFI**	**M-RFI**	**H-RFI**		
*Cutaneotrichosporon*	0.71 ± 0.02	14.49 ± 3.50	0.00	4.79	0.40
*Debaryomyces*	5.47 ± 0.90	2.45 ± 2.40	12.28 ± 1.90	2.89	0.38
*Hannaella*	0.00	7.40 ± 1.78	0.35 ± 0.05	3.40	0.39
*Kazachstania*	4.43 ± 1.03	0.00	1.98 ± 0.37	1.46	0.49
*Penicillium*	4.74 ± 0.40	6.12 ± 0.84	9.69 ± 0.96	1.79	0.53
*Ustilago*	4.30 ± 0.86	3.25 ± 0.04	2.91 ± 0.36	1.32	0.91
*Fusarium*	6.60 ± 4.41	5.10 ± 4.06	4.56 ± 1.27	1.12	0.76
*Alternaria*	5.09 ± 4.79	3.76 ± 3.17	0.97 ± 1.12	0.84	0.12
*Vishniacozyma*	1.51 ± 1.50	2.81 ± 3.70	1.72 ± 2.14	0.59	0.66
*Cladosporium*	6.36 ± 3.40	4.48 ± 2.78	5.58 ± 3.42	0.73	0.60
Others	60.79 ± 13.36	50.13 ± 27.64	59.96 ± 34.28	4.75	0.62

**Figure 4:**
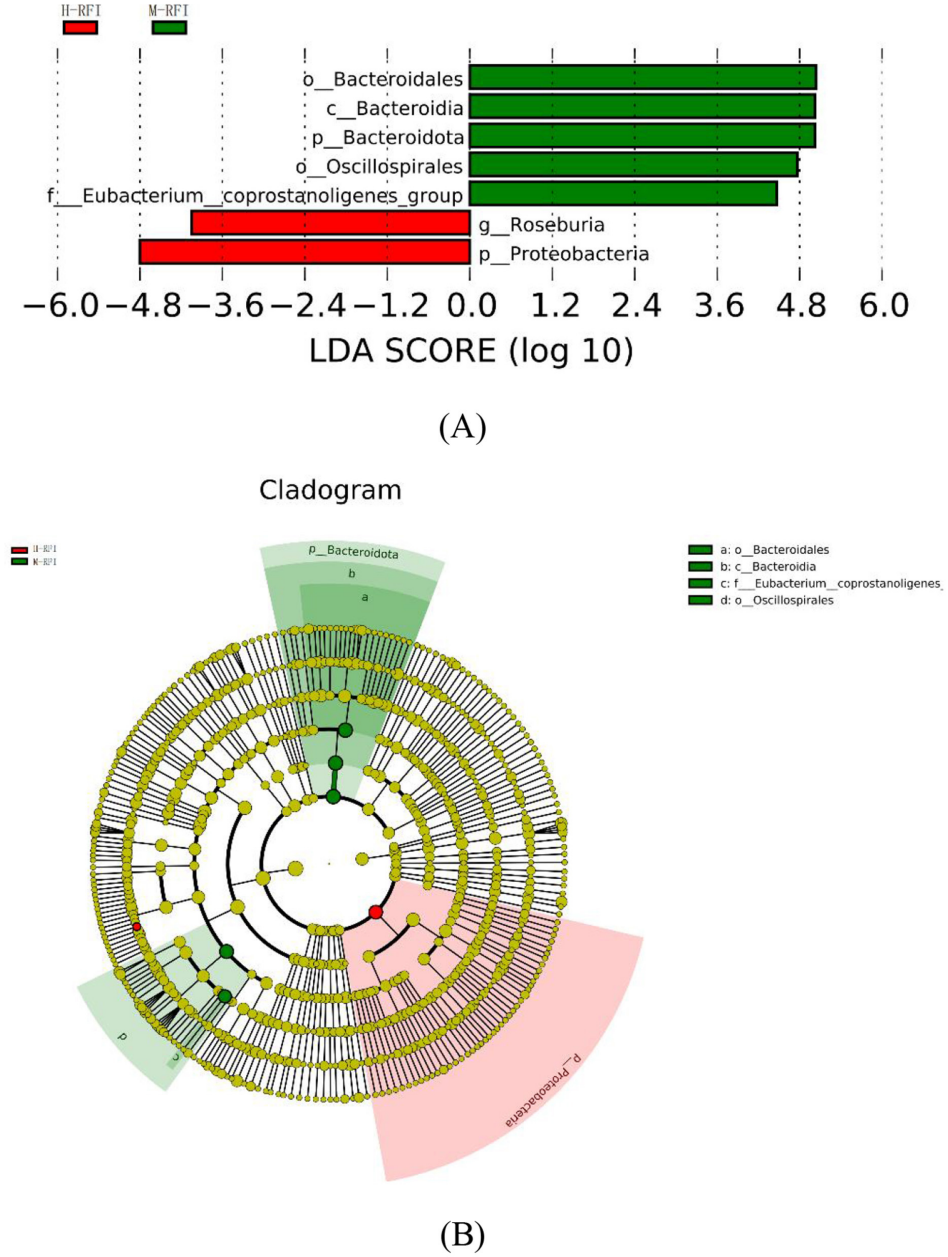
**(A, B)** LEfSe diagram of rumen digesta of male Dexin lambs with different RFIs.

As shown in Figure 4, there were seven species with LDA difference >4.0. The results show that the genera with the greatest impact on RFI on community structure are *p__Proteobacteria* and *g__Roseburia in H-RFI*, and *o__Bacteroidales, c__Bacteroidia, o__Oscillospirales, p__Bacteroidota*, and *f__Eubacterium__coprostanoligenes_group* in M-RFI. No differences were detected in fungi and L-RFI group.

**Figure 5:**
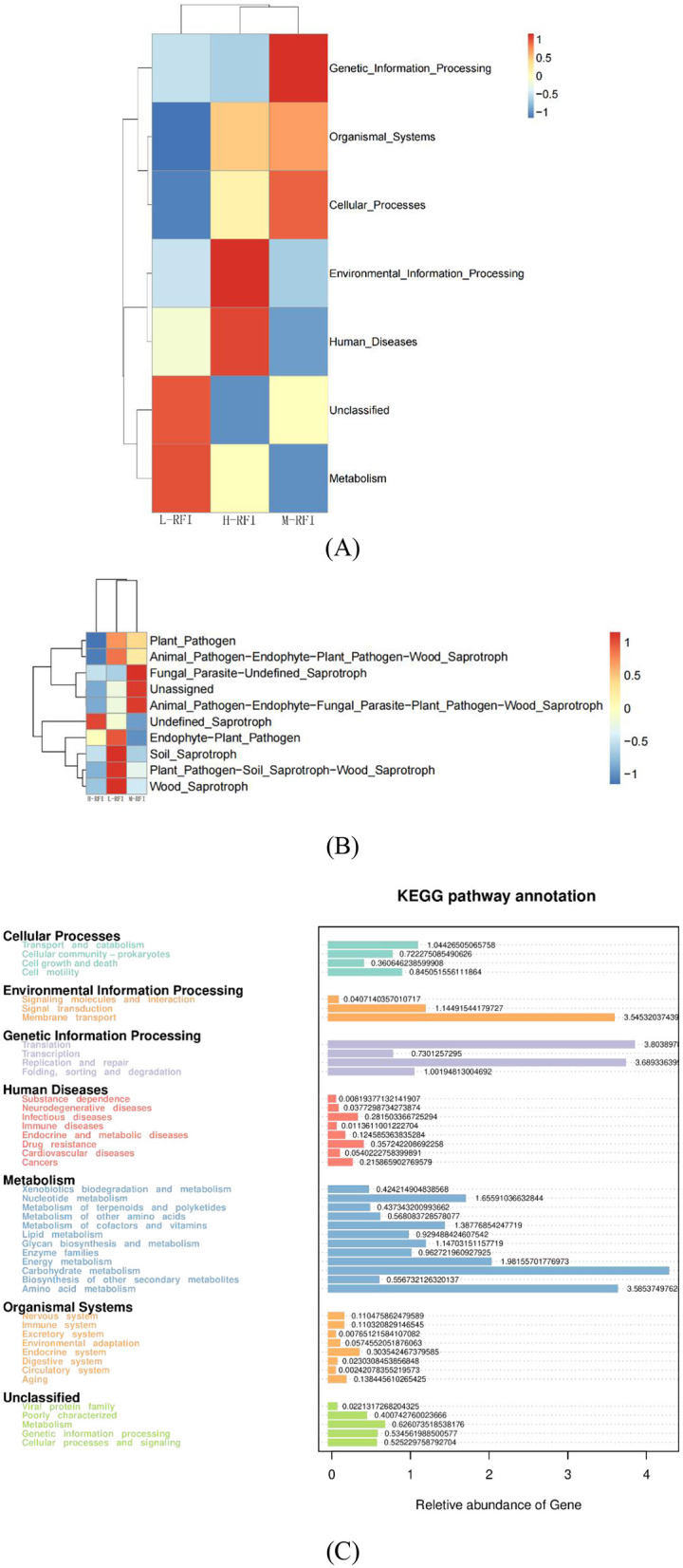
Diagram showing functions of predominant microbes in rumen fluid. Bacteria **(A, C)** and Fungi **(B)**.

It can be seen from Figures 5A, C that the bacteria identified in the different RFI groups are mainly involved in membrane transport, gene translation, carbohydrate metabolism, energy production, and amino acid metabolism. The main differences in fungi in rumen digesta occurred in the L-RFI group and included wood-digesting saprophytes, soil saprophytes, plant pathogens and endophytic plant pathogens. The H-RFI group contained unclassified saprophytes, while the M-RFI samples mainly included animal pathogens, unclassified, and undefined saprophytes (Figure 5B).

### 3.4 Relative bacterial populations in ileal digesta of male Dexin lambs with different RFIs

As shown in Supplementary Figure S3A, 1,781 OTUs were found in L-RFI, 1,195 in M-RFI, and 1,454 in H-RFI among the bacteria identified in ileum digesta samples of male Dexin lambs with different RFIs. Among them, there were 401 OTUs in common among all three groups, 578 OTUs in common between L-RFI and M-RFI, 652 OTUs in common between L-RFI and H-RFI, and 533 OTUs in common between H-RFI and M-RFI. With respect to the fungi, 496 OTUs were found in L-RFI, 354 OTUs were found in M-RFI, and 448 OTUs were found in H-RFI (Supplementary Figure S3B). Among them, L-RFI and H-RFI had 107 OTUs in common, L-RFI and M-RFI had 102, M-RFI and H-RFI had 103, and among all three groups there were 80 OTUs in common.

As shown in Supplementary Table S3, in the ileal digesta samples of male Dexin lambs with different RFIs, the bacterial there were no significant differences in Chao1, Simpson, and Simpson indices between any of the groups (*P* > 0.05). There were no significant differences in the Chao1, Shannon, and Simpson indices among the fungal groups (*P* > 0.05) (Supplementary Table S4).

As shown in Supplementary Figures S4A, B, the PCA plots of ileal chyme partially overlapped without obvious separation, indicating that the microbial communities in the ileal chyme samples were less diverse between and within groups.

**Figure 6:**
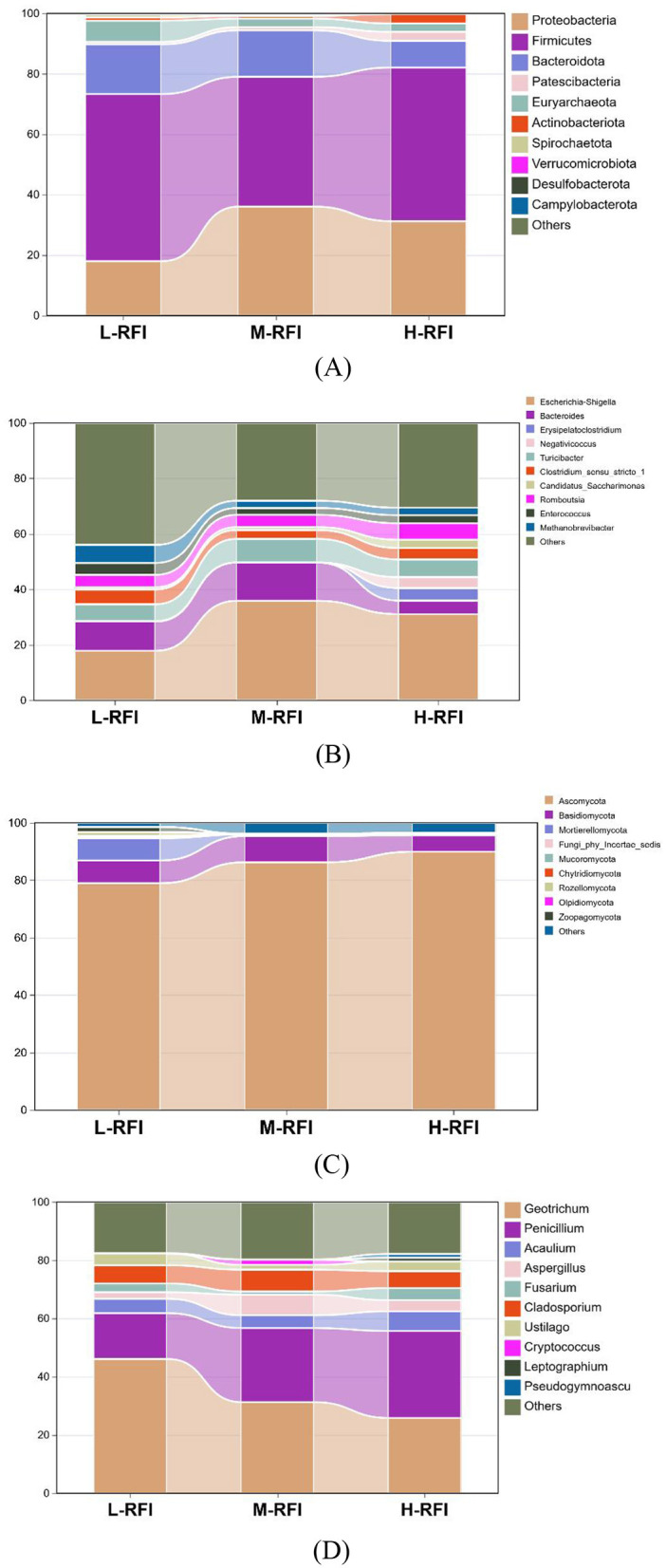
Histogram of ileal chyme microbiota in the different RFI groups. Bacterial phylum level **(A)**, Bacterial genus level **(B)**, Fungal phylum level **(C)**, and Fungal genus level **(D)**.

At the phylum level, *Firmicutes, Bacteroidota*, and *Proteobacteria* were the dominant phyla in ileal chyme, The Campylobacterota bacteria in the L-RFI group were significantly higher than those in the H-RFI group (*P* < 0.05), while there were no significant differences in the other bacteria (*P* > 0.05) (Figure 6A, Table 7). At the genus level, *Escherichia-Shigella, Bacteroides*, and *Erysipelatoclostridium* were the main dominant genera in the ileal chyme (Figure 6B, Table 8). For the *Methanobrevibacter* genus, L-RFI was significantly higher than M-RFI and H-RFI (*P* < 0.05), and there were no significant differences in the remaining genera (*P* > 0.05). At the fungal phylum level, the dominant phyla in ileal chyme samples were Ascomycota, Basidiomycota and *Mortierellomycota*, and there was no significant difference among the top ten dominant phyla (*P* > 0.05) (Figure 6C, Table 9). At the fungal genus level, *Geotrichum, Penicillium*, and *Cladosporium* were the dominant genera, and there was no significant difference among the top ten dominant genera (*P* > 0.05) (Figure 6D, Table 10).

**Table 7:** Relative abundance of ileal chyme bacteria at the phylum level in different RFI groups.

**Item**	**Group**			**SEM**	***P*-value**
	**L-RFI**	**M-RFI**	**H-RFI**		
*Proteobacteria*	18.00 ± 14.77	36.16 ± 13.58	31.27 ± 31.44	5.34	0.38
*Firmicutes*	55.31 ± 19.81	43.05 ± 16.25	50.87 ± 24.52	4.87	0.62
*Bacteroidota*	16.45 ± 12.25	15.42 ± 17.86	8.86 ± 7.25	3.02	0.55
*Patescibacteria*	0.82 ± 1.92	1.07 ± 1.25	2.98 ± 5.57	0.85	0.54
*Euryarchaeota*	6.98 ± 4.48	2.83 ± 2.43	2.78 ± 4.34	1.02	0.15
*Actinobacteriota*	1.07 ± 0.61	0.87 ± 0.70	3.11 ± 3.69	0.57	0.21
*Spirochaetota*	1.06 ± 1.45	0.42 ± 0.43	0.05 ± 0.05	0.06	0.18
*Verrucomicrobiota*	0.10 ± 0.19	0.05 ± 0.06	0.01 ± 0.02	0.03	0.44
*Desulfobacterota*	0.10 ± 0.16	0.002 ± 0.003	0.01 ± 0.01	0.03	0.17
*Campylobacterota*	0.07 ± 0.06^a^	0.03 ± 0.03^ab^	0.01 ± 0.01^b^	0.01	0.04
Others	0.03 ± 0.02	0.08 ± 0.08	0.05 ± 0.01	0.01	0.23

In the same row, values with no letter or the same letter superscripts mean no significant difference (*P* > 0.05), while with different small letter superscripts mean significant difference (*P* < 0.05).

**Table 8:** Relative abundance of ileal chyme bacteria at the genus level in the different RFI groups.

**Item**	**Group**			**SEM**	***P*-value**
	**L-RFI**	**M-RFI**	**H-RFI**		
*Escherichia-Shigella*	17.86 ± 14.80	35.89 ± 13.80	31.07 ± 31.46	5.35	0.38
*Bacteroides*	10.54 ± 11.36	13.77 ± 18.44	4.88 ± 3.21	2.89	0.62
*Erysipelatoclostridium*	0.14 ± 0.33	0.04 ± 0.08	4.52 ± 1.06	1.53	0.55
*Negativicoccus*	0.003 ± 0.005	0.0008 ± 0.001	3.92 ± 7.44	1.11	0.54
*Turicibacter*	6.11 ± 4.69	8.48 ± 6.58	6.39 ± 6.27	1.35	0.15
*Clostridium_sensu_stricto_1*	5.32 ± 6.56	3.22 ± 3.56	4.12 ± 4.11	1.15	0.21
*Candidatus_Saccharimonas*	0.82 ± 1.91	1.06 ± 1.24	2.96 ± 5.58	0.85	0.18
*Romboutsia*	4.43 ± 5.10	4.40 ± 3.11	5.96 ± 5.29	1.08	0.44
*Enterococcus*	4.30 ± 5.25	2.47 ± 1.97	2.91 ± 4.14	0.95	0.17
*Methanobrevibacter*	6.50 ± 4.72^a^	2.60 ± 2.28^b^	2.69 ± 4.32^b^	1.02	0.04
Others	43.97 ± 22.20	28.08 ± 12.62	30.56 ± 18.12	4.53	0.23

In the same row, values with no letter or the same letter superscripts mean no significant difference (*P* > 0.05), while with different small letter superscripts mean significant difference (*P* < 0.05).

**Table 9:** Relative abundance of fungal microbiota at the phylum level in ileal digesta from different RFI groups.

**Item**	**Group**			**SEM**	***P*-value**
	**L-RFI**	**M-RFI**	**H-RFI**		
Ascomycota	74.37 ± 5.21	86.28 ± 11.67	89.92 ± 3.38	5.16	0.46
Basidiomycota	7.46 ± 4.71	9.09 ± 5.51	5.79 ± 2.75	1.10	0.50
Mortierellomycota	7.31 ± 2.13	0.58 ± 1.14	0.68 ± 1.53	2.42	0.46
Fungi_phy_Incertae_sedis	0.31 ± 0.35	0.21 ± 0.20	0.15 ± 0.14	0.05	0.53
Mucoromycota	0.30 ± 0.10	0.02 ± 0.02	0.01 ± 0.01	0.08	0.25
Chytridiomycota	0.08 ± 0.01	0.00	0.00	0.02	0.35
Rozellomycota	1.33 ± 0.90	0.00	0.00	0.44	0.39
Olpidiomycota	0.04 ± 0.01	0.00	0.00	0.02	0.39
Zoopagomycota	1.61 ± 0.70	0.00	0.00	0.53	0.39
Others	1.37 ± 1.00	3.82 ± 6.78	3.45 ± 4.07	0.93	0.54

**Table 10:** Relative abundance of fungal microbiota at the genus level in ileal digesta from different RFI groups.

**Item**	**Group**			**SEM**	***P*-value**
	**L-RFI**	**M-RFI**	**H-RFI**		
*Geotrichum*	46.15 ± 8.75	31.27 ± 28.52	25.86 ± 31.96	6.08	0.37
*Penicillium*	15.75 ± 7.25	25.43 ± 11.56	29.84 ± 15.17	1.10	0.14
*Acaulium*	4.93 ± 3.18	4.40 ± 7.81	6.74 ± 8.75	2.42	0.84
*Aspergillus*	2.27 ± 0.93	7.06 ± 8.43	3.83 ± 2.75	1.19	0.28
*Fusarium*	3.02 ± 2.28	1.10 ± 0.90	4.15 ± 6.32	0.96	0.47
*Cladosporium*	6.22 ± 1.78	7.47 ± 3.42	5.81 ± 3.16	0.66	0.61
*Ustilago*	4.04 ± 2.79	1.66 ± 0.93	3.33 ± 3.27	0.64	0.34
*Cryptococcus*	0.00	1.78 ± 4.00	0.00	0.52	0.32
*Leptographium*	0.00	0.00	1.31 ± 3.20	0.46	0.42
*Pseudogymnoascus*	0.15 ± 0.03	0.09 ± 0.10	1.28 ± 2.35	0.35	0.26
Others	17.56 ± 5.10	19.69 ± 8.44	17.81 ± 6.59	1.52	0.85

**Figure 7:**
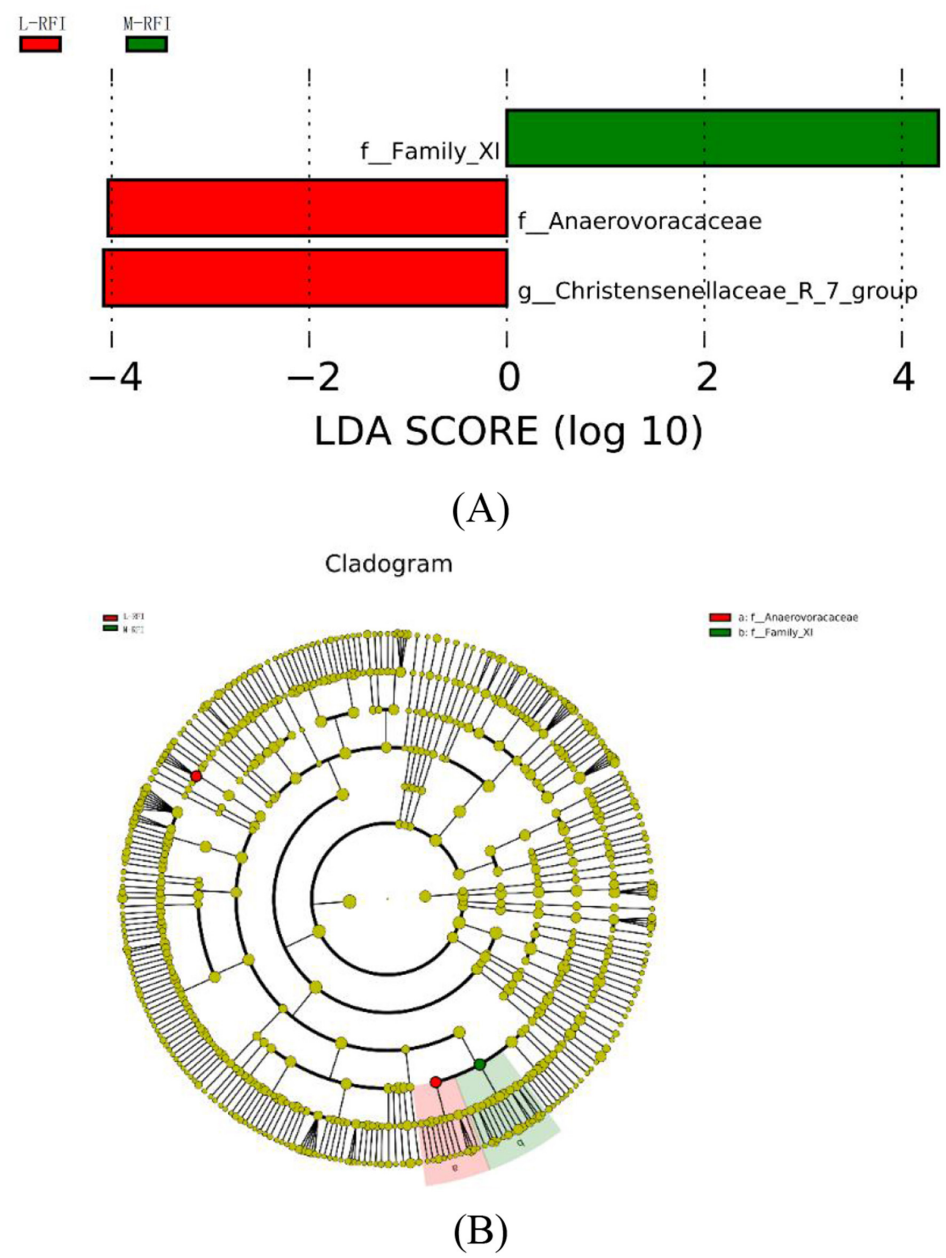
**(A, B)** LEfSe of ileum digesta from male Dexin lambs with different residual feed intake.

As shown in Figures 7A, B, there are three species with LDA values >4, indicating that the bacterial genera that have a greater impact on ileal chyme due to residual feed intake are *f-Family-Xi* in M-RFI*, f_Anaerovoracaceae* and *g_Christensenellaceae_R_7_group* in L-RFI. No differences were detected in fungi and H-RFI group.

**Figure 8:**
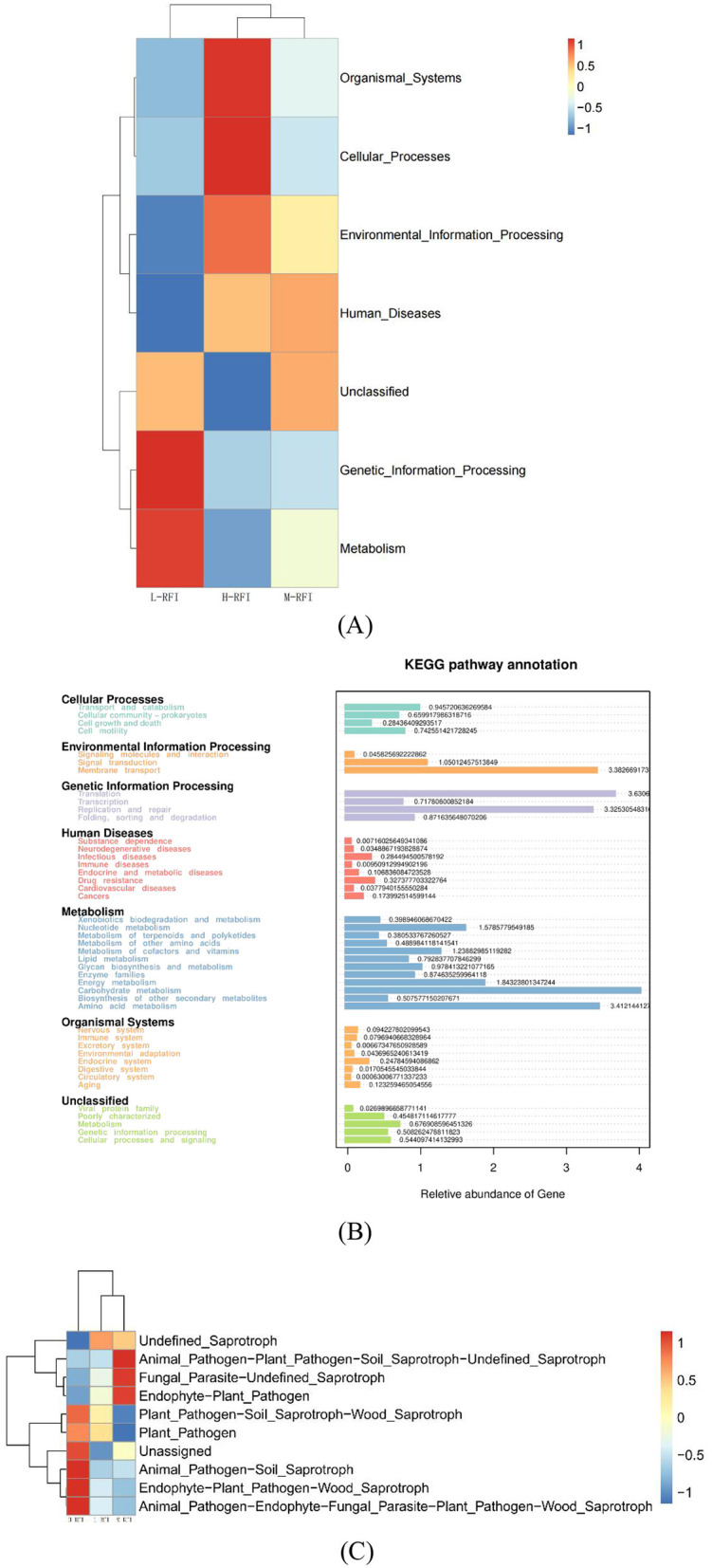
Prediction of ileum digesta functions of male Dexin lambs with different RFIs. Bacteria **(A, C)**, Fungi **(B)**.

The differentially expressed functions of ileal digesta bacteria from different RFI groups were mainly membrane transport, carbohydrate metabolism, and amino acid metabolism (Figures 8A, B). Among the ileal chyme fungi, the functional annotations of L-RFI and H-RFI fungi were quite different, mainly concentrated in plant pathogens, undefined animal pathogens, endophytic plant pathogens and undefined saprophytes (Figure 8C).

### 3.5 Differences in bacterial microbiota from rectal feces of male Dexin lambs with different RFIs

As shown in Supplementary Figure S5A, 979 OTUs were found in the samples of rectal feces from L-RFI lambs, 870 from M-RFI, and 1,454 from H-RFI. There were 293 OTUs in common among the three groups, 404 OTUs in common between L-RFI and M-RFI, 442 OTUs in common between L-RFI and H-RFI, and 425 OTUs in common between H-RFI and M-RFI. A total of 500 OTUs were found in the fungi of the rectal fecal samples from L-RFI, 1,170 OTUs from M-RFI, and 1,246 OTUs from H-RFI, of which 31 OTUs were found in common in all three groups, 101 OTUs in common in L-RFI and M-RFI, 99 OTUs in common in L-RFI and H-RFI, and 72 OTUs in common in H-RFI and M-RFI (Supplementary Figure S5B).

As shown in Supplementary Tables S5, S6, there were no significant differences in the Chao1, Shannon, and Simpson indices observed in any of the groups of rectal fecal samples (*P* > 0.05).

As shown in Supplementary Figure S6, there is a clear separation between L-RFI and H-RFI in the rectal feces' samples of male Dexin lambs with different RFI, indicating that the microbiota in the two groups are quite different.

**Figure 9:**
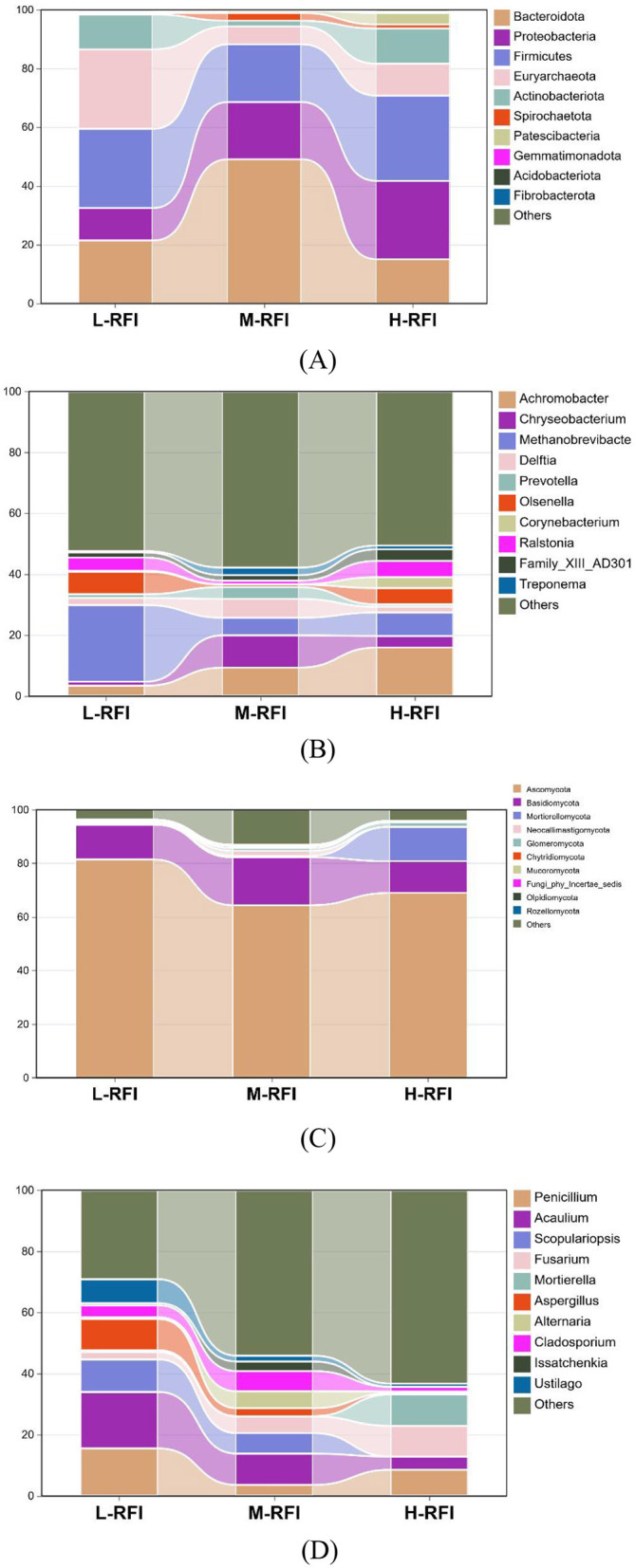
Column chart of rectal fecal microbiota in the different RFI groups. Bacterial phylum level **(A)**, Bacterial genus level **(B)**, Fungal phylum level **(C)**, and Fungal genus level **(D)**.

As shown in Figure 9A and Table 11, at the phylum level, *Euryarchaeota, Bacteroidia*, and *Firmicutes* were the main bacterial groups in rectal feces. The *Uryarchaeota* phylum in L-RFI was extremely significantly higher than that in M-RFI and H-RFI (*P* < 0.01), the *Actinobacteriota* phylum in L-RFI and H-RFI was significantly higher than that in M-RFI (*P* < 0.05), the *Patescibacteria* phylum in H-RFI was significantly higher than that in M-RFI and L-RFI (*P* < 0.05), but there were no significant differences in other phyla (*P* > 0.05). At the genus level, *Achromobacter, Chryseobacterium*, and *Methanobrevibacter* were the dominant bacterial genera in rectal feces (Figure 9B, Table 12). The abundance of *Methanobrevibacter* in L-RFI was significantly higher than in M-RFI and H-RFI (*P* < 0.05), and there were no significant differences in the other genera (*P* > 0.05). The dominant rectal fecal fungi at the phylum level were Ascomycota, Basidiomycota and *Mortierellomycota*, and there were no significant differences among the three groups in the top ten dominant phyla (Figure 9C, Table 13). At the genus level for rectal fecal fungi, *Penicillium, Acaulium*, and *Scopulariopsis* were the dominant genera (Figure 9D, Table 14). The levels of *Aspergillus* and *Ustilago* genera in the L-RFI group were significantly higher than those in the other two groups (*P* < 0.05), but those in the “other” group were significantly lower than those in the two RFI groups (*P* < 0.05).

**Table 11:** Relative abundance of bacteria at the phylum level in rectal feces from the different RFI groups.

**Item**	**Group**			**SEM**	***P*-value**
	**L-RFI**	**M-RFI**	**H-RFI**		
*Bacteroidota*	21.45 ± 0.67	49.08 ± 20.21	15.05 ± 12.87	6.57	0.50
*Proteobacteria*	11.03 ± 9.46	19.46 ± 27.75	26.66 ± 18.52	3.40	0.65
*Firmicutes*	26.96 ± 5.99	19.68 ± 13.11	29.03 ± 12.23	3.40	0.57
*Euryarchaeota*	27.04 ± 4.81^Aa^	6.09 ± 3.54^Bb^	10.88 ± 4.31^Bb^	3.39	0.002
*Actinobacteriota*	11.89 ± 4.04^a^	1.94 ± 1.35^b^	11.99 ± 6.14^a^	2.08	0.04
*Spirochaetota*	0.44 ± 0.51	2.65 ± 4.58	1.36 ± 2.26	0.92	0.67
*Patescibacteria*	0.58 ± 0.29^b^	0.83 ± 0.60^b^	3.91 ± 1.72^a^	0.62	0.02
*Gemmatimonadota*	0.01 ± 0.01	0.00	0.23 ± 0.40	0.08	0.43
*Acidobacteriota*	0.00	0.00	0.12 ± 0.22	0.04	0.42
*Fibrobacterota*	0.00	0.11 ± 0.19	0.02 ± 0.01	0.04	0.48
Others	0.58 ± 0.42	0.15 ± 0.12	0.74 ± 0.49	0.14	0.22

In the same row, values with no letter or the same letter superscripts mean no significant difference (*P* > 0.05), while with different small letter superscripts mean significant difference (*P* < 0.05), and with different capital letter superscripts mean extremely significant difference (*P* < 0.01).

**Table 12:** Relative abundance of bacteria at the genus level in rectal feces from the different RFI groups.

**Item**	**Group**			**SEM**	***P*-value**
	**L-RFI**	**M-RFI**	**H-RFI**		
*Achromobacter*	3.38 ± 2.47	9.33 ± 15.70	15.92 ± 14.32	4.00	0.50
*Chryseobacterium*	1.34 ± 1.28	10.61 ± 16.73	3.80 ± 3.22	3.40	0.52
*Methanobrevibacter*	25.16 ± 4.10^Aa^	5.76 ± 3.44^Bb^	7.7 ± 2.19^Bb^	3.23	0.001
*Delftia*	2.32 ± 1.98	6.20 ± 10.36	1.89 ± 2.07	1.92	0.66
*Prevotella*	1.23 ± 1.78	3.86 ± 0.64	0.78 ± 0.81	1.21	0.60
*Olsenella*	7.37 ± 3.36	0.81 ± 0.61	5.35 ± 3.16	1.24	0.06
*Corynebacterium*	0.33 ± 0.10	0.10 ± 0.12	3.58 ± 5.86	1.12	0.42
*Ralstonia*	4.34 ± 4.65	1.22 ± 1.73	5.33 ± 3.74	1.21	0.40
*Family_XIII_AD3011_group*	1.68 ± 0.61	1.80 ± 1.76	3.82 ± 3.47	0.74	0.47
*Treponema*	0.42 ± 0.48	2.47 ± 4.27	1.22 ± 2.03	0.84	0.67
Others	52.43 ± 6.03	57.84 ± 36.29	50.60 ± 20.26	7.08	0.93

In the same row, values with no letter or the same letter superscripts mean no significant difference (*P* > 0.05), while with different small letter superscripts mean significant difference (*P* < 0.05), and with different capital letter superscripts mean extremely significant difference (*P* < 0.01).

**Table 13:** Relative abundance of rectal fecal fungi at the phylum level in different RFI groups.

**Item**	**Group**			**SEM**	***P*-value**
	**L-RFI**	**M-RFI**	**H-RFI**		
*Ascomycota*	81.58 ± 21.55	64.94 ± 18.62	68.93 ± 11.22	0.05	0.36
*Basidiomycota*	13.02 ± 11.10	17.99 ± 3.52	11.84 ± 4.30	0.03	0.73
*Mortierellomycota*	0.63 ± 0.04	0.59 ± 0.30	12.82 ± 8.13	0.03	0.09
*Neocallimastigomycota*	0.00	1.92 ± 1.22	0.00	0.01	0.42
*Glomeromycota*	0.00	1.00 ± 0.80	1.68 ± 1.10	0.003	0.09
*Chytridiomycota*	0.20 ± 0.11	0.74 ± 0.50	0.46 ± 0.38	0.002	0.48
*Mucoromycota*	0.75 ± 0.60	0.01 ± 0.01	0.00	0.002	0.08
*Fungi_phy_Incertae_sedis*	0.28 ± 0.20	0.49 ± 0.34	0.02 ± 0.01	0.001	0.24
*Olpidiomycota*	0.00	0.00	0.08 ± 0.01	0.002	0.08
*Rozellomycota*	0.01 ± 0.01	0.02 ± 0.01	0.04 ± 0.02	0.0001	0.60
Others	3.71 ± 2.73	13.19 ± 10.32	4.12 ± 4.11	0.03	0.49

**Table 14:** Relative abundance of rectal fecal fungi at the genus level in the different RFI groups.

**Item**	**Group**			**SEM**	***P*-value**
	**L-RFI**	**M-RFI**	**H-RFI**		
*Penicillium*	15.52 ± 6.11	3.59 ± 4.21	8.57 ± 4.12	0.03	0.39
*Acaulium*	18.46 ± 10.23	10.24 ± 8.89	4.25 ± 3.21	0.03	0.22
*Scopulariopsis*	10.67 ± 8.81	6.7 ± 5.12	0.00	0.03	0.33
*Fusarium*	2.37 ± 1.54	5.46 ± 4.32	10.07 ± 7.58	0.02	0.33
*Mortierella*	0.62 ± 0.50	0.08 ± 0.02	10.31 ± 7.11	0.02	0.08
*Aspergillus*	10.21 ± 4.13^a^	2.62 ± 1.11^b^	0.44 ± 0.31^b^	0.02	0.03
*Alternaria*	0.58 ± 0.40	5.53 ± 4.31	0.5 ± 0.30	0.13	0.24
*Cladosporium*	3.9 ± 2.49	6.6 ± 5.78	1.5 ± 1.11	0.13	0.32
*Issatchenkia*	0.7 ± 0.33	3.15 ± 1.31	0.02 ± 0.01	0.01	0.48
*Ustilago*	7.82 ± 4.57^a^	1.89 ± 2.10^b^	1.05 ± 1.22^b^	0.12	0.02
Others	29.16 ± 11.43^b^	54.15 ± 13.21^a^	63.29 ± 44.1^a^	0.06	0.02

In the same row, values with no letter or the same letter superscripts mean no significant difference (*P* > 0.05), while with different small letter superscripts mean significant difference (*P* < 0.05).

**Figure 10:**
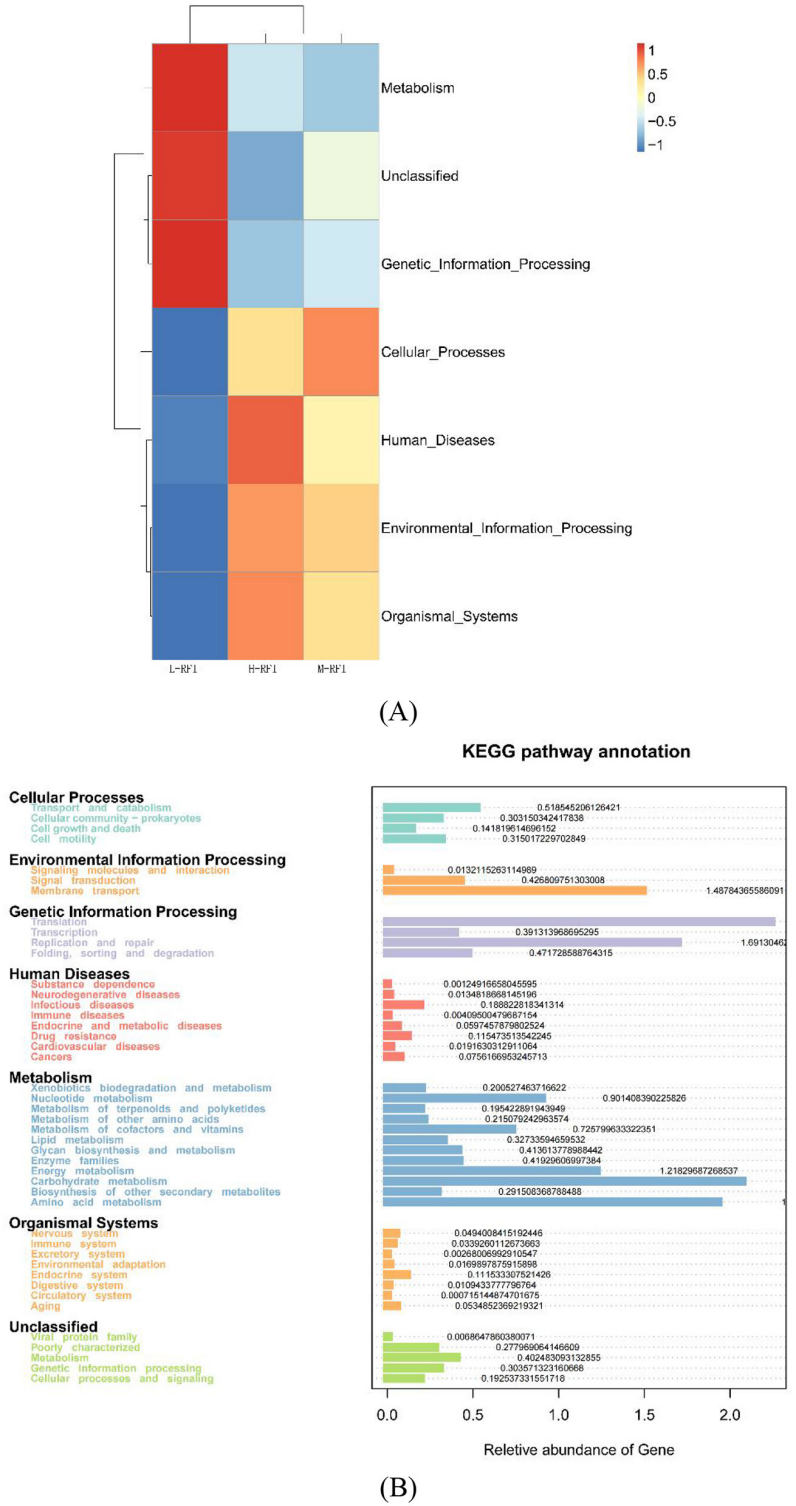
**(A, B)** Prediction of rectal fecal microbial functions of male Dexin lambs with different RFIs.

As shown in Figures 10A, B, the expression differences in the microbial functions of rectal fecal bacteria are mainly concentrated in membrane transport, energy production, carbohydrate and amino acid metabolism.

## 4 Discussion

The rumen is the main site for ruminants to receive feed, water and saliva. It is a humid environment with a favorable temperature of 36–40°C, which makes it an excellent place for microbial growth and reproduction (Nagaraja, 2016). A large number of studies have shown that the various microbial communities in the rumen work synergistically to convert substances such as cellulose and hemicellulose into volatile fatty acids, and convert the nitrogen produced by dietary degradation into microbial proteins that are absorbed and utilized by the host (Dijkstra, 1994).^1^ The apparent digestibility of dry matter, crude protein, neutral detergent fiber and other insdicators can accurately mirror a sheep's ability to digest and absorb nutrients (da Cruz et al., 2021). In a study on the effect of RFI on apparent digestibility, Bonilha et al. (2017) found that the digestibility of neutral detergent fiber and total digestible nutrients in Neruda cattle in the H-RFI group was significantly lower than in the L-RFI group. The research results of Arce-Recinos et al. (2021) on growing beef cattle showed that the digestibility of dry matter, crude protein, neutral detergent fiber and acid detergent fiber of L-RFI cattle was 4%−5% higher than that of H-RFI cattle, similar to the results in this study. The apparent digestibility of dry matter, crude protein, neutral detergent fiber and acid detergent fiber of L-RFI male Dexin lambs was higher than in the H-RFI group, indicating that apparent digestibility may be one of the reasons why some sheep show higher feed efficiency. In this experimental study, no difference in rumen volatile fatty acids was found among the three groups of sheep, which is similar to the findings of two studies by Arce-Recinos et al. (2022) and Zhang et al. (2022). This may be due to the fact that the feed substrates were exactly the same, making it difficult to establish a difference in the volatile fatty acid content.

In the OTU results of this experiment, the fungal and bacterial microbiota detected in the L-RFI group were greater than those in the M-RFI and H-RFI groups, suggesting that an enrichment of beneficial microbes could enable individuals to have higher environmental adaptability and stress resistance. There were no significant differences in the Chao1, Shannon, and Simpson indices of bacteria and fungi in the rumen digesta, ileum digesta, and rectal feces samples among the three groups. The PCoA plots did not show significant differences in the microorganisms from the three groups of sheep, which is consistent with the results of Pinnell et al. (2022) on the rumen of Holstein cows. This may be because the RFI only affects individual microbial types and not the overall microbial community.

Ruminants rely on the abundant microbial communities in their digestive tract to digest feed and convert it into nutrients that are easily absorbed. The most abundant gut bacteria are from the *Firmicutes* and *Bacteroidota* phyla (Hernández et al., 2022). Recent progress in microbial research indicates that *Bacteroidota* can produce large amount of glycoside hydrolases, which can effectively degrade nutrients such as cellulose, pectin and starch, and plant polysaccharides in the rumen (Milligan et al., 1967). *Firmicutes* are mainly polysaccharide-degrading microorganisms in the rumen, but recent studies indicate that Firmicutes can produce biotin, playing a major role in cellulose degradation, VFA production, and metabolism (Chen et al., 2011; Zhang et al., 2021). In this study, at the bacterial phylum level, the dominant phyla of bacteria in rumen digesta, ileum digesta and rectal feces were *Firmicutes, Bacteroidota*, and *Proteobacteria*. The rumen bacteria results were consistent with the results of Liu et al. (2022) on the rumen microbiota of Hu sheep with different RFIs. The rumen of healthy ruminants is characterized by the dominance of anaerobic bacteria of *Firmicutes* and *Bacteroidota*. The results of ileal digesta were consistent with those of Elolimy et al. (2020), with *Firmicutes* and *Bacteroidota* as the main bacterial communities, and the abundance of *Firmicutes* and *Bacteroidota* in the L-RFI group was higher than that in the H-RFI group. The rectal feces results were similar to those of Elolimy (Avguštin et al., 1994), with the abundance of *Bacteroidota* in cattle with L-RFI being higher than that in cattle with H-RFI. At the bacterial genus level, the genera with greatest abundance in rumen chyme were *Bacteroides, Rikenellaceae_RC9_gut_group*, and *Prevotella*_7. Among them, the abundance of *Bacteroides* in the L-RFI group was higher than that in the H-RFI group, and the abundance of *Rikenellaceae_RC9_gut_group* was lower than that in the H-RFI group. The *Bacteroides* genus has been shown to effectively degrade plant cell wall polysaccharides and improve fiber utilization (Liu et al., 2023). The specific role of the *Rikenellaceae*_RC9_gut_group genus is still unclear, and it has so far only been shown to be related to butyrate and propionate metabolism (He et al., 2022). In the ileal digesta, *Escherichia-Shigella, Bacteroides*, and *Turicibacter* were the main genera. The abundance of *Bacteroides* in L-RFI lambs was higher than that in the H-RFI group, while the abundance of *Escherichia-Shigella* was lower than that in the H-RFI group. *Escherichia-Shigella* is a harmful bacterium that may cause bacterial dysentery (Carmichael et al., 2024). *Methanobrevibacter* is a methanogen. Although it was not the main genus in terms of abundance in the ileal digesta, the data showed that its numbers in the L-RFI group were significantly higher than in the H-RFI group. This was contrary to the results of Mia (Friedman et al., 2017), which showed that the ileal digesta of L-RFI male Dexin lambs contained more methanogens. In our results, the abundance of methanogens in the three parts of the L-RFI group was higher than that in the H-RFI group. This may be a result of the sheep production model and feed type. However, recent microbiological research indicates that methanogens are of great significance to the early intestinal microbial colonization of ruminants. They can effectively reduce the H_2_ produced by the fermentation and decomposition of plant fibers in the digestive tract, reduce the hydrogen partial pressure, and improve the body's hydrogen nutrition pathway (Menetrey et al., 2021). However, the mechanistic details and regulatory pathways need to be verified by subsequent studies. In rectal feces, *Achromobacter, Chryseobacterium*, and *Methanobrevibacter* were the main genera. The abundance of *Methanobrevibacter* in the L-RFI group was significantly higher than that in the H-RFI group, while the abundance of *Achromobacter* and *Chryseobacterium* was lower than in the H-RFI group. *Achromobacter* is a conditionally pathogenic bacterium that can cause urinary tract infections under certain conditions (Page et al., 2019). *Chryseobacterium* has a strong ability to digest collagen and can cause disease in the body (Gordon and Phillips, 1998). Through the abundance analysis of bacterial microbiota under the three RFI conditions, we can conclude that L-RFI sheep achieve higher digestion efficiency of feed nutrients by having a greater abundance of *Firmicutes* and *Bacteroidetes* in the digestive tract. This echoes the results of apparent digestibility in this study, indicating that L-RFI sheep have a higher efficiency of decomposition and absorption of feed nutrients through an enriched population of microorganisms. The increased abundance of pathogenic bacteria detected in the H-RFI group may be a result of differences in their digestive tract microbiota, which makes them less adaptable and resistant than the L-RFI sheep, which is consistent with our OTU results.

There are a large number of anaerobic fungi in the GIT of ruminants. Previous studies have generally concluded that these fungi exist in the animal body in the form of zoospores, which produce highly active, fiber-degrading enzymes, and play a major role in the digestion of fibrous plant material (Hagen et al., 2020). It is increasingly recognized that fungi can optimize rumen fermentation, enhance nutrient availability, and promote intestinal health. Anaerobic fungi degrade plant cell walls through both enzymatic reactions and physical means targeting fibers that are difficult for bacteria to degrade (Hartinger and Zebeli, 2021). In addition to fiber degradation in the rumen, 5% to 10% of carbohydrate degradation occurs in the hindgut, indicating that fiber degradation by anaerobic fungi can occur over the entire digestive tract (McAllister et al., 1994). It has been proven that Basidiomycota and Ascomycota can produce mycelial hyphae, which can penetrate the silica cuticle produced on the surface of forage through stomata and damaged parts of the dermis, thereby promoting more efficient digestion of plant fiber (Chesson and Forsberg, 1997; Akin et al., 1983). Basidiomycota and Ascomycota are aerobic fungi with the highest relative abundance, based on sampling of rumen chyme. Within 2 h of eating, the oxygen concentration in the rumen is sufficient for the survival of Ascomycota. As aerobic fungi multiply, the oxygen content gradually decreases. This process causes the rumen to turn into an anaerobic environment, and anaerobic fungi and bacteria begin to multiply in large numbers (McAllister et al., 1993; Wang et al., 2023). Mortierellomycota is a type of saprophytic fungus that is widely distributed in the digestive tract of ruminants and has the ability to efficiently decompose lignin (Li et al., 2022).

In this experiment, at the phylum level, the main fungal phyla in rumen and ileum digesta were Basidiomycota, Ascomycota and Mortierellomycota, among which Basidiomycota and Mortierellomycota were more abundant in L-RFI sheep, and Ascomycota was more abundant in the H-RFI group. At the genus level, *Cladosporium, Fusarium*, and *Debaryomyces* were the dominant genera in rumen digesta. Current microbiological studies have shown that Cladosporium fungi can produce lipases, proteases, urease, and chitinase, which can help the host digest corn-rich diets (Messini et al., 2017). Fusarium can produce biomass that is broken down and transformed by the animal rumen; however, *in vivo* research on the effects of *Fusarium* on nutrient digestibility and rumen function is lacking and the details of the specific mechanism of action are still unclear (Rotter et al., 2022). *Debaryomyces* has emerged as a potentially valuable probiotic. Its cell wall and the polyamines it produces have been shown to stimulate immunity, regulate the microbiome, and improve digestive function (Angulo et al., 2020). However, the abundance of *Debaryomyces* in the H-RFI group was higher than that in the L-RFI group.

At the genus level in the ileal digesta, the dominant fungal genera were *Geotrichum, Penicillium*, and *Cladosporium*. *Geotrichum* are believed to have the potential to be useful probiotics, which can increase the production of acetic acid and propionic acid and the ratio of propionic acid to acetic acid in ruminants; their effectiveness is higher than that of traditional brewer's yeast (Long et al., 2013). The cellulase produced by *Penicillium* can effectively increase the content of oleic acid, linoleic acid and linolenic acid in milk, and can also affect the fat yield and unsaturated fatty acid content of milk (Azzaz et al., 2021). In the results of this study, although there was no significant difference in the abundance of *Geotrichum* in the ileal digesta among the three groups, the abundance in the L-RFI group was 21% higher than that in the H-RFI group, which may be related to the improvement in feed efficiency. Aspergillus can secrete cellulase and protease to improve the digestibility of the feed (Guo et al., 2022). *Ustilago* plays a role in lignin degradation and participates in the fermentation of feed (Barraza et al., 2021). In our experiments, *Aspergillus* and *Ustilago* were significantly more abundant in the L-RFI group than in the other two groups, suggesting that *Aspergillus* may play a major role in improving feed efficiency.

LefSE is a high-dimensional biomarker mining tool based on the LDA algorithm, which is used to identify significantly characterized microorganisms (Chang et al., 2022). In the results of this experiment, when LDS > 4.0, the characteristic bacteria were only found in the rumen and ileum digesta. Among them, the g__*Roseburia* genus found in the H-RFI group in rumen chyme is believed to affect the core populations and produce ketones that affect host development (Chai et al., 2024). P_Proteobacteria may parasitize the phylum Proteobacteria in rumen fluid and rumen epithelium, and Proteobacteria may oxidize ammonia and methane on the surface of the rumen epithelium (Mitsumori et al., 2002). In the ileal chyme, the f_Anaerovoracaceae bacteria found in the L-RFI group can utilize a variety of types of organic matter as carbon sources and participate in the fermentation of plant polysaccharides in the GIT (Boutard et al., 2014). The g_Christensenellaceae_R_7_group is currently thought to be a new type of probiotic that can effectively improve the growth performance and meat quality of ruminants. It plays an important role in the degradation of carbohydrates and amino acids into acetate and ammonia, respectively (Chen et al., 2020; Wei et al., 2022). The LEfSE results suggest that there are differences in some probiotics in the digestive tracts of sheep in the L- and H-RFI groups, which may be the main reason for the differences in feed utilization efficiency. The Proteobacteria found in the rumen chyme will compete for H ions with methanogens in the digestive tract, which may be one of the reasons for the differences in the abundance of methanogens among the three groups.

The KEGG (Kyoto Encyclopedia of Genes and Genomes) pathway analysis is a software module that builds a manually curated pathway graph representing the current knowledge on biological networks under defined conditions in a specific organism. The pathway diagrams are graphical representations of the networks of interacting molecules responsible for specific cellular functions (Zhang and Wiemann, 2009). In our study, the rumen chyme KEGG diagram showed that the differential metabolic pathways between L-RFI and H-RFI were mainly concentrated in Cellular Process (L-RFI), Metabolism (L-RFI), and Environmental Information Processing (H-RFI). Among these, the Cellular Process pathway is mainly concentrated in the Transport and Catabolism pathway, the Metabolism pathway is mainly related to carbohydrate metabolism, amino acid metabolism and energy production pathways, and the Environmental Information Processing metabolic pathway was mainly concentrated in the Membrane Transport pathway. This is similar to the research results of Zhang et al. (2021) and Zhou et al. (2023). The ileal chyme KEGG map shows that the pathway differences are mainly Cellular Process (L-RFI), Metabolism (L-RFI), Genetic Information Processing (L-RFI), and Environmental Information Processing (H-RFI). Among these, the Cellular-Process pathway was mainly concentrated in cell motility, the Genetic Information Processing pathway was mainly concentrated in replication and repair, the Metabolism pathway was mainly concentrated in carbohydrate metabolism, amino acid metabolism and energy production, and the Environmental Information Processing metabolic pathway was mainly concentrated in membrane transport. In the rectal fecal KEGG map, only the Environmental Information Processing pathway and the Human Diseases pathway were overexpressed in the H-RFI group. The Environmental Information Processing pathway was mainly concentrated in the membrane transport pathway, and the Human Diseases pathway was mainly concentrated in the endocrine system pathway. This is quite different from the research results of Elolimy et al. (2020) on Holstein cattle, which may be related to the breed, production mode and gender.

Overall, the KEGG analysis indicated that the overexpression in the L-RFI group was mainly concentrated in carbohydrate metabolism, amino acid metabolism and energy production, which may be related to the high abundance of *Bacteroidetes* and *Firmicutes* in the microbiota of L-RFI sheep, which could effectively improve the conversion and absorption of nutrients such as cellulose and amino acids. In the LEfSE results, some differentially expressed microbial metabolites had the function of promoting carbohydrate metabolism and amino acid decomposition and conversion, which may be related to the differences in KEGG metabolic pathways.

In the KEGG diagram of fungi from the rumen and ileum digesta, the main differences observed in the fungi in rumen fluid were wood saprophytes (L-RFI), soil saprophytes (L-RFI), plant pathogens and endophytic plant pathogens (L-RFI), and unclassified saprophytes (H-RFI). The functions of ileal chyme fungi are mainly concentrated in undefined (H-RFI), endophytic plant pathogens (H-RFI) and animal pathogens (H-RFI). Given the current limitations in the KEGG functional annotation of fungi, the specific metabolic pathways should be further explored.

## 5 Conclusions

According to the results of this study, different RFI did not significantly affect the overall digestive tract microbial community of Dexin male lambs. Its main mechanism of action may be to improve feed efficiency by changing the abundance of certain beneficial bacteria.

## Data Availability

The raw data supporting the conclusions of this article will be made available by the authors, without undue reservation.

## Footnotes

^1^*Study on rumen microbial function and mammary amino acid metabolism differences and their mechanisms in dairy cows with different feed efficiency*. Zhejiang University.
