# Supplementary material for: Effect of differences in residual feed intake on gastrointestinal microbiota of Dexin fine-wool meat sheep
Source: Front Microbiol. 2024 Dec 20;15:1482017. doi: 10.3389/fmicb.2024.1482017 (PMC11697150; doi:10.3389/fmicb.2024.1482017)
Supplement: Supplementary file 1 [file Data_Sheet_1.docx]

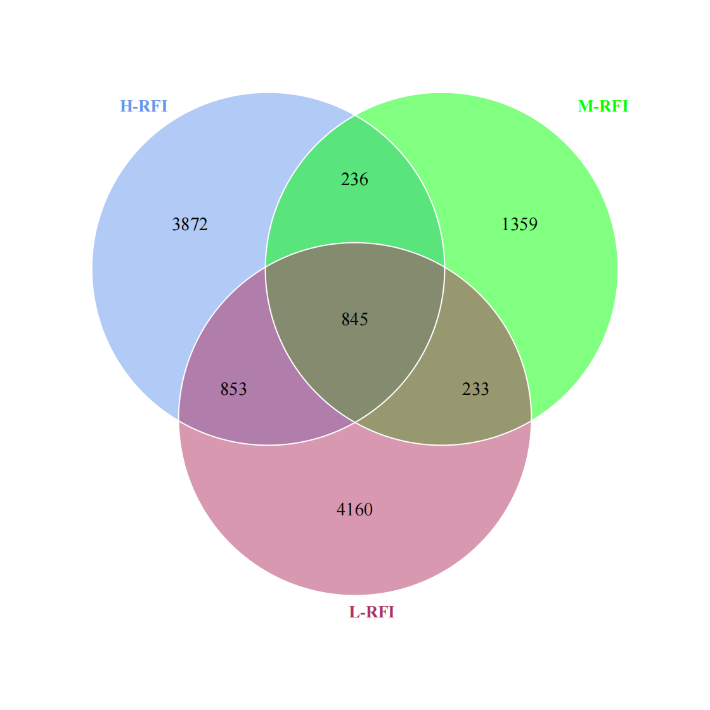

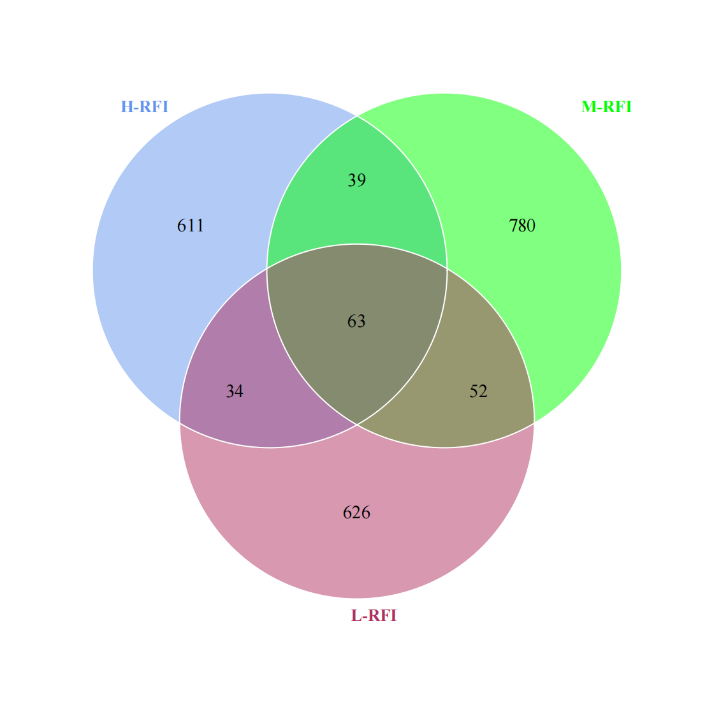


(A) (B)

**Figure S1.** Venn diagram overlaps of OTUs from male Dexin lambs with different RFIs. Bacteria (A). Fungi (B).

**Table S1.** Bacterial alpha diversity analysis of rumen digesta samples from different RFI groups.

| **Item** | **Group** | | | **SEM** | ***P*-value** |
| --- | --- | --- | --- | --- | --- |
|  | L-RFI | M-RFI | H-RFI |  |  |
| Chao1 | 1275±780.79 | 741.15±267.76 | 1299.26±890.11 | 175.21 | 0.39 |
| Shannon | 6.98±1.51 | 6.03±0.73 | 6.60±1.54 | 0.32 | 0.53 |
| Simpson | 0.94±0.05 | 0.94±0.03 | 0.92±0.08 | 0.01 | 0.75 |

**Table S2.** Fungal alpha diversity analysis of rumen digesta samples from different RFI groups.

| **Item** | **Group** | | | **SEM** | ***P*-value** |
| --- | --- | --- | --- | --- | --- |
|  | L-RFI | M-RFI | H-RFI |  |  |
| Chao1 | 144.24±43.45 | 203.94±93.81 | 132.56±32.66 | 15.53 | 0.15 |
| Shannon | 5.34±1.13 | 5.88±1.17 | 5.79±0.81 | 0.33 | 0.65 |
| Simpson | 0.91±0.12 | 0.94±0.08 | 0.96±0.03 | 0.21 | 0.59 |


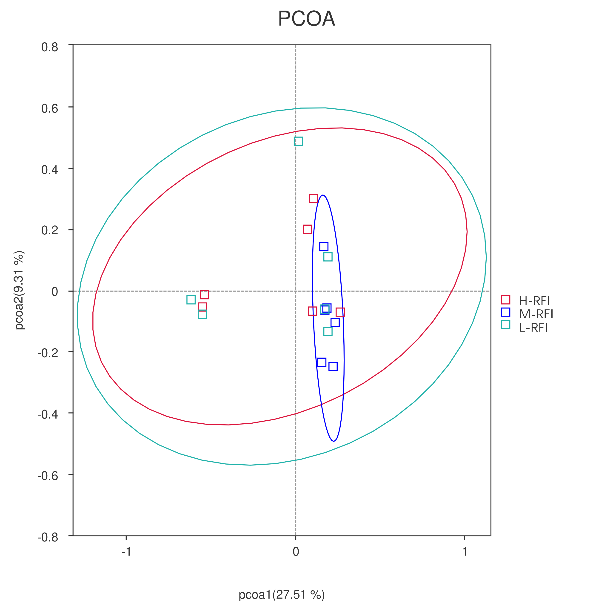

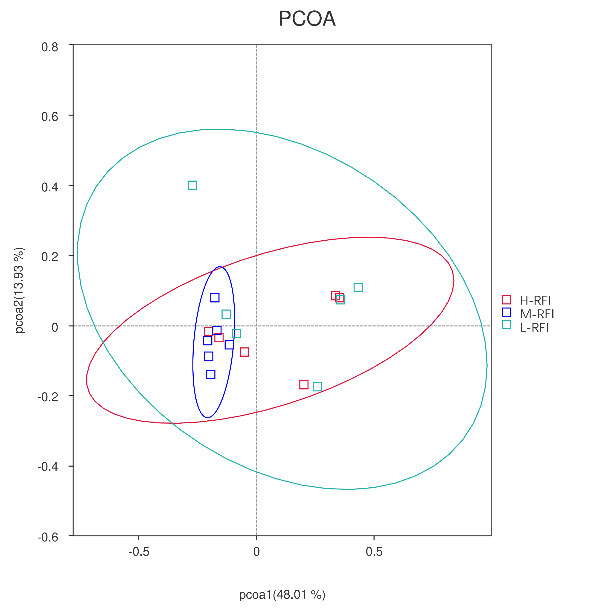


(A) (B)

**Figure S2.** PCoA chart of rumen digesta microorganisms of male Dexin lambs with different RFIs. Bacteria (A). Fungi(B).


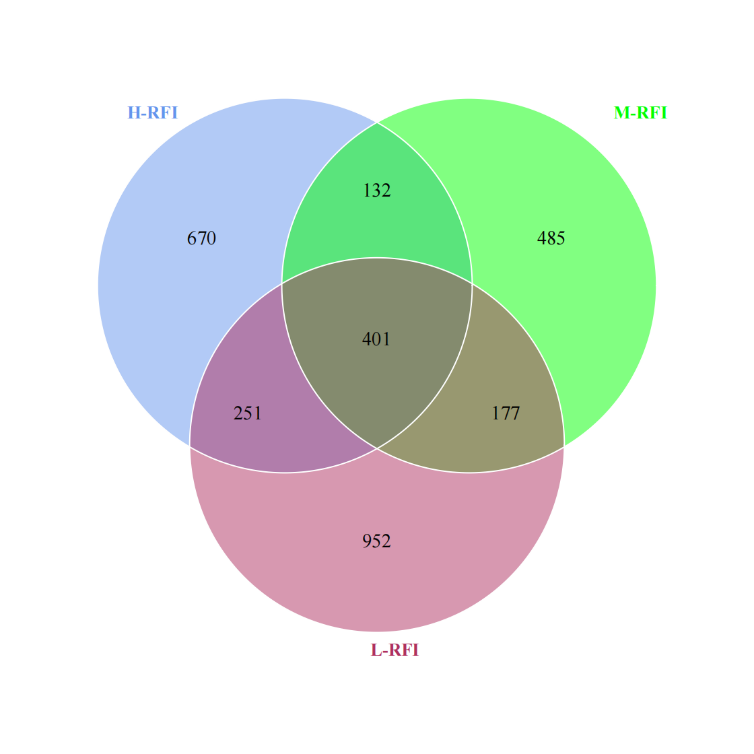

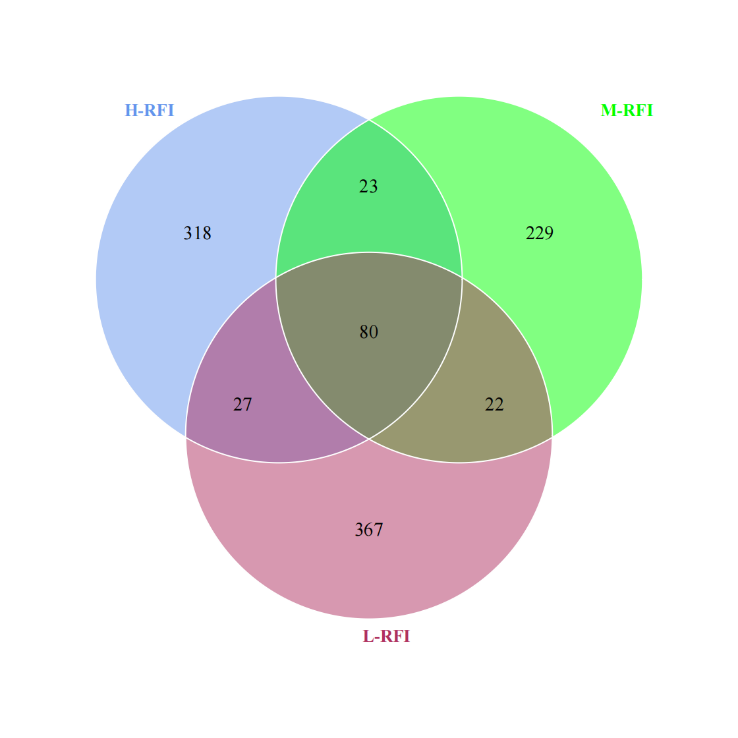


(A) (B)

**Figure S3.** Venn diagram of overlapping OTUs in microorganisms in ileal digesta from different RFI groups. Bacteria (A), Fungi (B).

**Table S3.** Alpha diversity analysis of bacteria in ileal chyme samples from the different RFI groups.

| **Item** | **Group** | | | **SEM** | ***P*-value** |
| --- | --- | --- | --- | --- | --- |
|  | L-RFI | M-RFI | H-RFI |  |  |
| Chao1 | 573.17±232.62 | 386.41±148.85 | 452.30±134.88 | 44.98 | 0.25 |
| Shannon | 5.57±1.45 | 4.12±0.79 | 4.42±1.50 | 0.33 | 0.18 |
| Simpson | 0.91±0.07 | 0.82±0.06 | 0.82±0.22 | 0.03 | 0.45 |

**Table S4.** Alpha diversity analysis of fungi in ileal chyme samples from the different RFI groups.

| **Item** | **Group** | | | **SEM** | ***P*-value** |
| --- | --- | --- | --- | --- | --- |
|  | L-RFI | M-RFI | H-RFI |  |  |
| Chao1 | 121.52±26.59 | 93.54±24.46 | 105.56±24.09 | 6.36 | 0.21 |
| Shannon | 3.96±0.43 | 3.85±0.30 | 4.01±0.81 | 0.13 | 0.90 |
| Simpson | 0.82±0.06 | 0.83±0.06 | 0.83±0.08 | 0.02 | 0.97 |


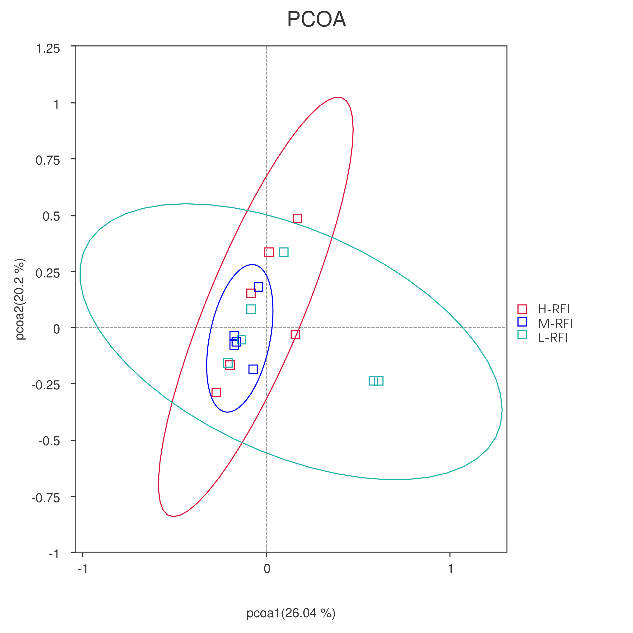

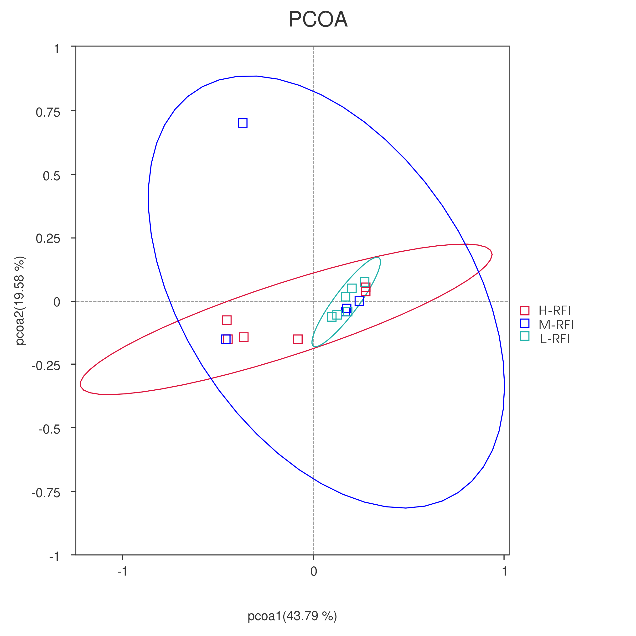


(A) (B)

**Figure S4.** PCoA diagram of microorganisms in ileal digesta of male Dexin lambs with different RFIs. Bacteria (A), Fungi (B).


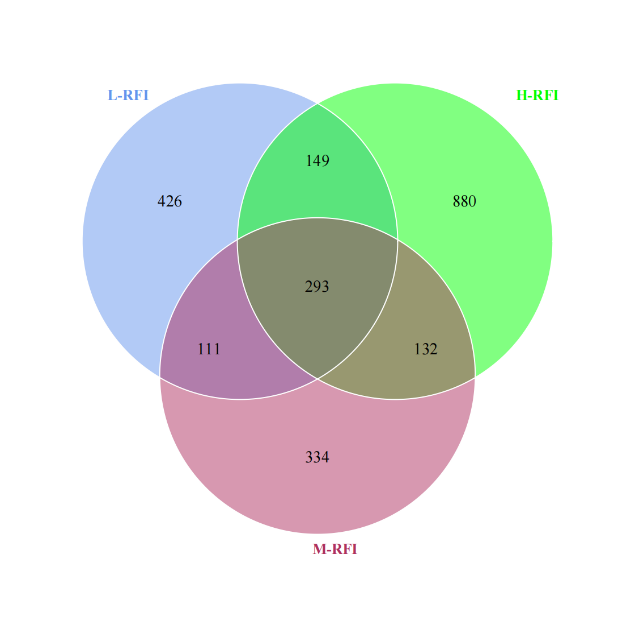

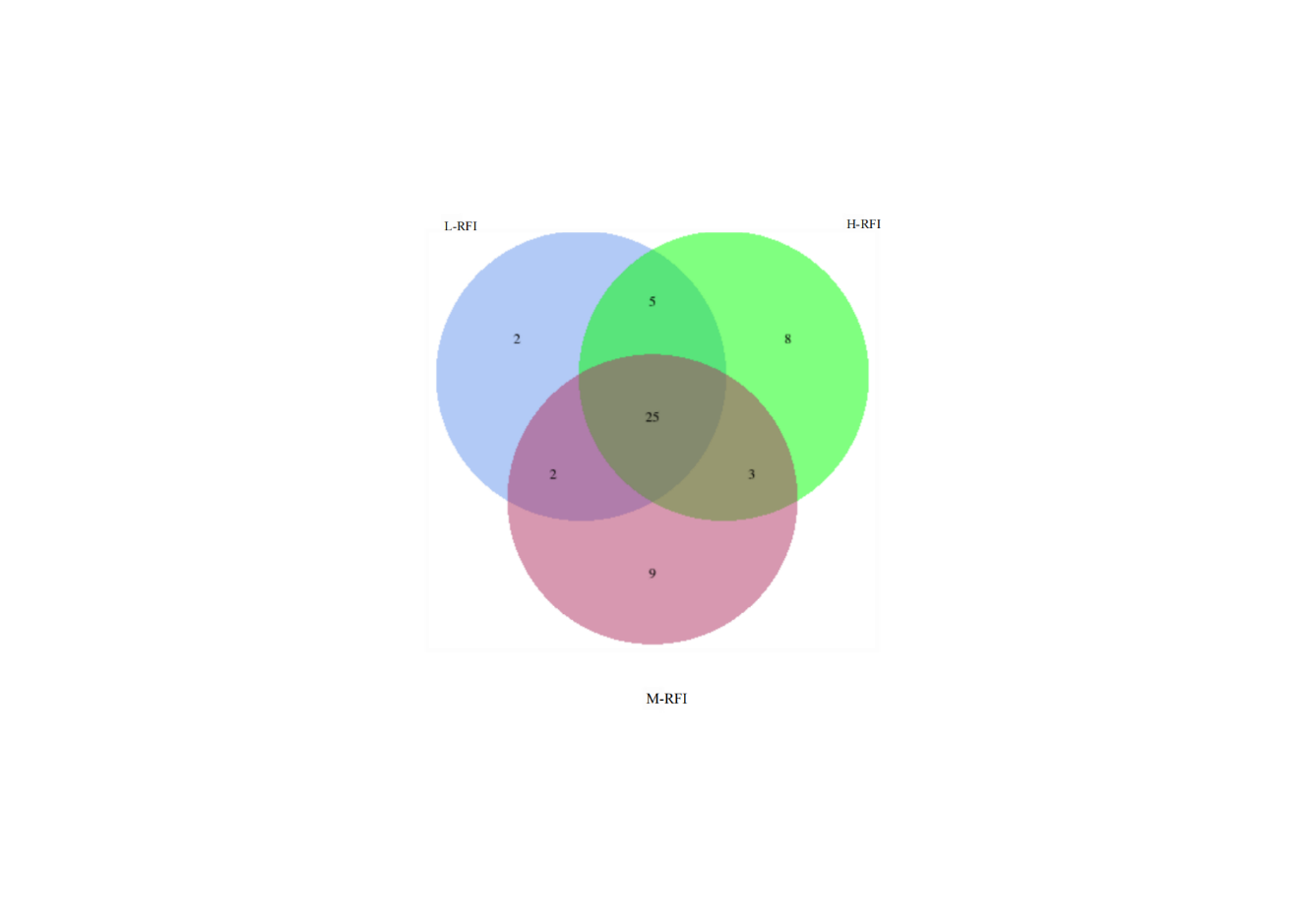


(A) (B)

**Figure S5.** Venn diagram of overlapping rectal fecal bacterial microorganisms in the different RFI groups. Bacteria(A), Fungi(B)

**Table S5.** Analysis of microbial alpha diversity of rectal fecal bacteria from different RFI groups.

| **Item** | **Group** | | | **SEM** | ***P*-value** |
| --- | --- | --- | --- | --- | --- |
|  | L-RFI | M-RFI | H-RFI |  |  |
| Chao1 | 479.67±149.24 | 324.79±100.62 | 644.38±263.77 | 84.89 | 0.35 |
| Shannon | 5.34±0.25 | 4.59±2.10 | 6.21±1.31 | 0.47 | 0.44 |
| Simpson | 0.92±0.02 | 0.85±0.12 | 0.94±0.05 | 0.02 | 0.36 |

**Table S6.** Analysis of microbial alpha diversity of rectal fecal fungi from different RFI groups.

| **Item** | **Group** | | | **SEM** | ***P*-value** |
| --- | --- | --- | --- | --- | --- |
|  | L-RFI | M-RFI | H-RFI |  |  |
| Chao1 | 317.71±131.92 | 454.22±408.52 | 606.26±468.56 | 113.81 | 0.65 |
| Shannon | 5.56±0.50 | 6.03±0.82 | 6.25±1.26 | 0.27 | 0.62 |
| Simpson | 0.94±0.01 | 0.95±0.01 | 0.96±0.01 | 0.04 | 0.40 |


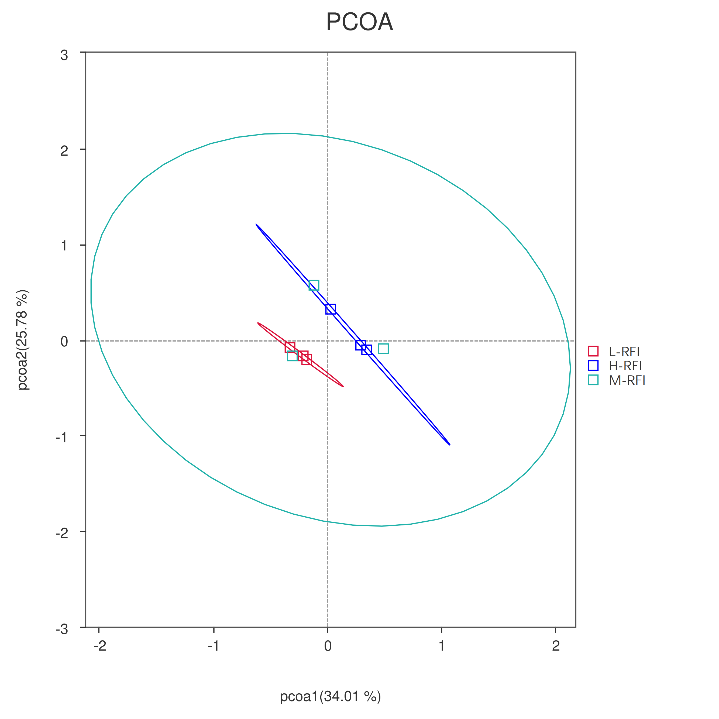


**Figure S6.** PCA plot of rectal fecal microorganisms from the different RFI groups.
